# B-type natriuretic peptide-guided therapy for heart failure (HF): a systematic review and meta-analysis of individual participant data (IPD) and aggregate data

**DOI:** 10.1186/s13643-018-0776-8

**Published:** 2018-07-31

**Authors:** Maria Pufulete, Rachel Maishman, Lucy Dabner, Julian P. T. Higgins, Chris A. Rogers, Mark Dayer, John MacLeod, Sarah Purdy, William Hollingworth, Morten Schou, Manuel Anguita-Sanchez, Patric Karlström, Michael Kleiner Shochat, Theresa McDonagh, Angus K. Nightingale, Barnaby C. Reeves

**Affiliations:** 1 Clinical Trials and Evaluation Unit, School of Clinical Sciences, University of Bristol, Level 7, Bristol Royal Infirmary, Queen’s Building, Bristol, BS2 8HW UK; 2 School of Social and Community Medicine, University of Bristol, Bristol, UK; 3 Department of Cardiology, Taunton and Somerset NHS Foundation Trust, Taunton, UK; 4 Herlev and Gentofte University Hospital, Herlev, DK-2730 Copenhagen, Denmark; 5 Agencia de Investigación de la Sociedad Española de Cardiología, Madrid, Spain; 6 Division of Cardiology, Department of Medicine, County Hospital Ryhov, Jönköping, Sweden; 7 Heart Institute, Hillel Yaffe Medical Center, Hadera, Israel; 8 Cardiovascular Division, King’s College Hospital, King’s College London, Denmark Hill, London, SE5 9RS UK; 9 Department of Cardiology, Bristol Heart Institute, Bristol Royal Infirmary, Bristol, BS2 8HW UK

**Keywords:** Heart failure, B-type natriuretic peptide, Systematic review, IPD meta-analysis

## Abstract

**Background:**

We estimated the effectiveness of serial B-type natriuretic peptide (BNP) blood testing to guide up-titration of medication compared with symptom-guided up-titration of medication in patients with heart failure (HF).

**Methods:**

Systematic review and meta-analysis of randomised controlled trials (RCTs). We searched: MEDLINE (Ovid) 1950 to 9/06/2016; Embase (Ovid), 1980 to 2016 week 23; the Cochrane Library; ISI Web of Science (Citations Index and Conference Proceedings). The primary outcome was all-cause mortality; secondary outcomes were death related to HF, cardiovascular death, all-cause hospital admission, hospital admission for HF, adverse events, and quality of life. IPD were sought from all RCTs identified. Random-effects meta-analyses (two-stage) were used to estimate hazard ratios (HR) and confidence intervals (CIs) across RCTs, including HR estimates from published reports of studies that did not provide IPD. We estimated treatment-by-covariate interactions for age, gender, New York Heart Association (NYHA) class, HF type; diabetes status and baseline BNP subgroups. Dichotomous outcomes were analysed using random-effects odds ratio (OR) with 95% CI.

**Results:**

We identified 14 eligible RCTs, five providing IPD. BNP-guided therapy reduced the hazard of hospital admission for HF by 19% (13 RCTs, HR 0.81, 95% CI 0.68 to 0.98) but not all-cause mortality (13 RCTs; HR 0.87, 95% CI 0.75 to 1.01) or cardiovascular mortality (5 RCTs; OR 0.88, 95% CI 0.67 to 1.16). For all-cause mortality, there was a significant interaction between treatment strategy and age (*p* = 0.034, 11 RCTs; HR 0.70, 95% CI 0.53–0.92, patients < 75 years old and HR 1.07, 95% CI 0.84–1.37, patients ≥ 75 years old); ejection fraction (*p* = 0.026, 11 RCTs; HR 0.84, 95% CI 0.71–0.99, patients with heart failure with reduced ejection fraction (HFrEF); and HR 1.33, 95% CI 0.83–2.11, patients with heart failure with preserved ejection fraction (HFpEF)). Adverse events were significantly more frequent with BNP-guided therapy vs. symptom-guided therapy (5 RCTs; OR 1.29, 95% CI 1.04 to 1.60).

**Conclusion:**

BNP-guided therapy did not reduce mortality but reduced HF hospitalisation. The overall quality of the evidence varied from low to very low. The relevance of these findings to unselected patients, particularly those managed by community generalists, are unclear.

**Systematic review registration:**

PROSPERO CRD42013005335

**Electronic supplementary material:**

The online version of this article (10.1186/s13643-018-0776-8) contains supplementary material, which is available to authorized users.

## Background

Heart failure (HF) affects over 500,000 people in the UK. Despite advances in medical treatment and evidence-based guidelines, patients continue to have high morbidity and poor life expectancy [1]. Many patients are not treated according to guidelines and do not receive optimal doses of available medications [2]. Clinicians sometimes find it difficult to recognise the early stages of worsening HF and are reluctant to increase doses of medications because of concerns about side effects such as renal failure and hypotension. Recently, biomarkers such as natriuretic peptides (B-type natriuretic peptide, BNP; or the N-terminal part of the precursor peptide of BNP, N-terminal pro-B-type natriuretic peptide, NT-proBNP, collectively referred to here as BNP), have been used as a more objective means of assessing HF severity and to prompt more appropriate titration of HF therapies.

Several randomised controlled trials (RCTs) have assessed whether using serial BNP tests to guide up-titration of medication improves clinical outcomes compared with symptom-guided therapy. The RCTs were heterogeneous in design. Most used a BNP-lowering strategy, where a BNP target was set (a single target for all patients or an individualised target) and HF medications were intensified to lower or maintain BNP at the pre-specified target. A few used a BNP-monitoring strategy, where the treating clinician was allowed to intensify HF medications using serial BNP measurements but no BNP target was set. Data from RCTs using a BNP-lowering strategy have been pooled in six aggregate data meta-analyses [3–8], one individual participant data (IPD) meta-analysis [9] All of these analyses showed that patients in the BNP-lowering group had better outcomes.

We conducted a systematic review and meta-analysis of IPD and aggregate data including all RCTs, regardless of BNP-guiding strategy [10]. Specific objectives were to estimate the effect of BNP-guided therapy on clinical outcomes; to estimate the extent of effect modification for key outcomes in specific subgroups; and to quantify the extent to which improved outcomes are explained by up-titration of medication and/or reduction in BNP levels. In this paper, we present an update of our meta-analysis to include data from the Guide-IT RCT [11], the largest RCT to date (894 patients), which planned to recruit 1100 patients but was terminated early because of futility.

## Methods

The protocol for the meta-analysis has been published previously [12]. The study population was all patients aged over > 18 years who were being treated for HF in primary or secondary care BNP-guided therapy or symptom-guided therapy. The primary outcome was all-cause mortality; secondary outcomes were death related to HF, cardiovascular death, all-cause hospitalisation, HF hospitalisation, adverse events, and quality of life.

### Search methods for identification of studies

The search strategy is shown in Additional file 1: Appendix 1. We searched the following electronic databases: MEDLINE (Ovid) 1950 to 6 September 2016; Embase (Ovid), 1980 to 2016, week 23; the Cochrane Library; ISI Web of Science (Citations Index and Conference Proceedings). We also searched the World Health Organization International Clinical Trials Registry Platform (WHO ICTRP; http://apps.who.int/trialsearch/) and Current Controlled Trials (http://www.isrctn.com/) to identify trials in progress. We reviewed reference lists of all full-text papers and also searched grey literature (http://www.opengrey.eu/ and Google Scholar).

### Study selection

Two review authors (MP and LD) independently triaged the titles and abstracts identified by the search and assessed the full text of all studies identified as relevant to the review. Differences in assessment by were resolved through discussion with a third author (RM). No language restriction was applied.

### Establishing the collaboration

Corresponding authors of eligible RCTs were invited to join the collaboration and were sent the IPD meta-analysis protocol with a cover letter explaining the study.

### Quality assessment

Two review authors (MP and LD) independently assessed the risk of bias (in accordance with recent Cochrane Collaboration guidelines [13]) in each included RCT. For blinding and incomplete outcome data, risk of bias was assessed separately for pre-specified outcome domains (all-cause mortality, cause-specific mortality, adverse events, and quality of life). For incomplete outcome data and selective outcome reporting, risk of bias was assessed only in RCTs that contributed aggregate data.

### Data collection and checking

IPD were collated into a single database. All datasets were checked for consistency against the original publication reports and discrepancies were discussed and clarified with authors via email. Where authors did not provide clarification, we documented assumptions that were made regarding the data.

### Statistical analysis

Meta-analysis was carried out if > 2 RCTs reported data on the outcome of interest. All analyses were performed on an intention-to-treat basis. Hazard ratios (HR) were estimated using Cox regression modelling for each RCT. For RCTs that did not provide IPD, HR estimates from published reports [14] were combined with HR estimates derived from the IPD. The HRs were combined across RCTs using random-effects meta-analysis (two stage model results using the generic inverse-variance method) [15], and consistency of findings across studies was assessed using the *I*^2^ test statistic. Fixed-effects meta-analysis was also performed as a secondary analysis. Subgroup effects were determined by estimating treatment-by-covariate interaction terms for each RCT and combining the HRs across RCTs as for the main effects [16]. Covariates defining subgroups were age (< 75 vs. ≥ 75 years); gender; New York Heart Association (NYHA) class (class I/II vs class III/IV); type of HF (reduced ejection fraction, HFrEF, vs. preserved ejection fraction, HFpEF, based on LVEF, < 40% in studies providing IPD and < 45% in studies providing aggregate data); diabetes status, BNP level (≤ vs. > median at baseline across all RCT participants, with separate medians calculated for RCTs that reported BNP and NTpro-BNP; cause of HF (ischaemic/non-ischaemic); previous atrial fibrillation; body mass index; systolic blood pressure. The age cut-off was chosen for consistency with other studies in elderly HF populations and to allow easy comparison with the meta-analysis by Troughton et al. [9]. For the LVEF cut-off, we used the lower limit of normal LVEF (40%) used in clinical practice. This threshold of 40% was pre-specified by the study authors, although for the aggregate data studies, we had to use the cut-off of 45% specified by the researchers of the existing IPD meta-analysis [17]. As for the main analysis, HR estimates from published reports [14] were combined with HR estimates derived from the IPD. We calculated interactions when these were not reported from subgroup-specific HRs with 95% confidence intervals (CI) from studies using aggregate data and pooled them with interactions from additional RCTs which had contributed IPD for this study. For cardiovascular mortality and adverse events, we calculated odds ratios (OR) and 95% CI in each trial and pooled these across RCTs using random effects meta-analyses. We assessed the certainty of the evidence across each outcome measure using the GRADE approach (risk of bias, consistency of effect, imprecision, indirectness, and publication bias) (http://www.gradeworkinggroup.org/.).

The relationship between the size of the treatment effect and the change in BNP values was investigated by plotting the ratio of change in BNP values (calculated using the formula below) against the hazard rate for each study with data available.

$$ \frac{\exp \left\{\ln \left(\mathrm{median}\ \mathrm{BNP}\ \mathrm{at}\ \mathrm{end}\ \mathrm{of}\ \mathrm{follow}-\mathrm{up}\ \mathrm{in}\ \mathrm{BNP}-\mathrm{guided}\ \mathrm{therapy}\ \mathrm{group}\right)-\ln \left(\mathrm{median}\ \mathrm{BNP}\ \mathrm{at}\ \mathrm{baseline}\ \mathrm{in}\ \mathrm{BNP}-\mathrm{guided}\ \mathrm{therapy}\ \mathrm{group}\right)\right\}}{\exp \left\{\ln \left(\mathrm{median}\ \mathrm{BNP}\ \mathrm{at}\ \mathrm{end}\ \mathrm{of}\ \mathrm{follow}-\mathrm{up}\ \mathrm{in}\ \mathrm{symptom}-\mathrm{guided}\ \mathrm{therapy}\ \mathrm{group}\right)-\ln \left(\mathrm{median}\ \mathrm{BNP}\ \mathrm{at}\ \mathrm{baseline}\ \mathrm{in}\ \mathrm{symptom}-\mathrm{guided}\ \mathrm{therapy}\ \mathrm{group}\right)\right\}} $$

For the three studies providing IPD data, the ratio of change was also calculated using the patient-specific change from baseline; after logarithmic transformation of all BNP values, the median change from baseline was calculated in each treatment group, and the ratio of the exponents of medians was calculated. All but two aggregate data studies provided median BNP values in their published report. For the two that did not (Christchurch Pilot and Signal-HF), we used the summary statistic reported (see Table 3). All analyses were conducted using Stata, v14.0, using the ‘ipdmetan’ command [18].

### Sensitivity analysis

The following sensitivity analyses were conducted: restricting the analysis to RCTs that defined a BNP target; and restricting the analysis to RCTs with good allocation concealment, since this has been shown to be an important source of bias in RCT.

### Checking for publication and data availability bias

Funnel plots were used to investigate association between the precision of the effect size and effect size (which could be due to publication bias or ‘small study effects’) [19], including and excluding RCTs for which IPD were unavailable. We included funnel plots only if a sufficient number of studies (more than 10) were available for each outcome.

## Results

**Fig. 1:**
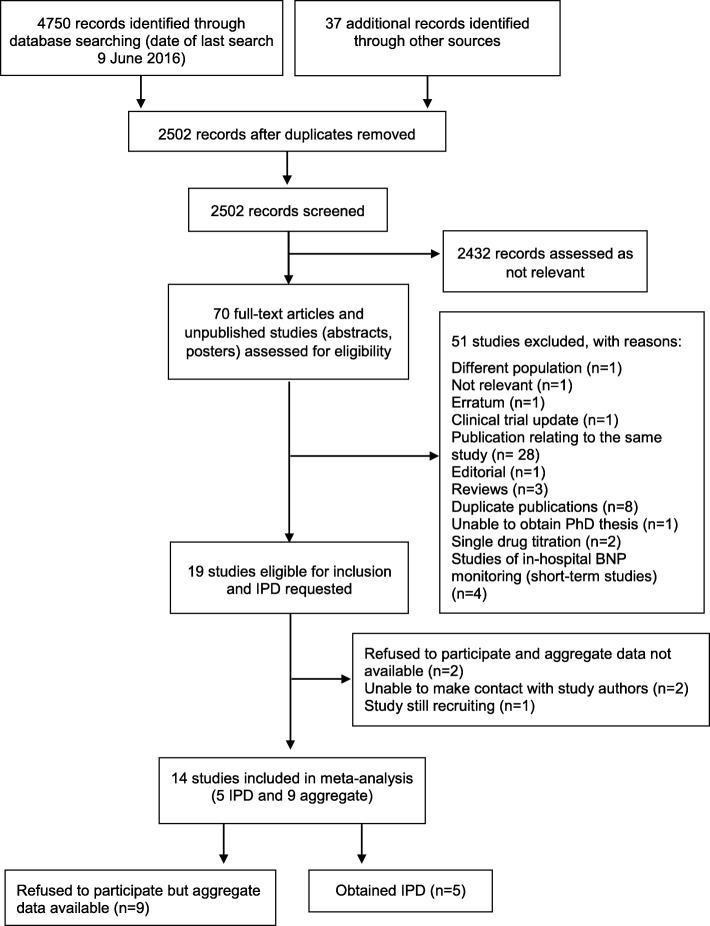
PRISMA flow diagram

Figure 1 shows the flow of studies through the review process. Full-text screening of 70 articles and unpublished studies identified 19 studies eligible for inclusion for which IPD were requested. Of these, 14 studies were included in the meta-analysis (5 IPD and 9 aggregate).

Table 1 shows the characteristics of the included RCTs. Of the 14 RCTs included in the meta-analysis, eight were conducted in Europe [20–27], two in New Zealand [28, 29], three in North America [11, 30, 31], and one in Israel [32]. One RCT (Time-CHF) published results separately for HFrEF [26] and HFpEF [33]. Only one RCT [25] (Signal-HF, Sweden) was conducted in primary care; the other 13 were conducted in hospital HF clinics, with most of these recruiting patients during or immediately after hospitalisation for HF. Twelve RCTs used a BNP-lowering strategy [11, 20–26, 28–31] and two used a BNP-monitoring strategy [27, 32]. Of the 12 RCTs that used a BNP-lowering strategy, nine set a single target (BNP 100–300 pg/ml; NT-proBNP 400–2200 pg/ml [11, 20, 21, 23, 24, 26, 28–30]), two of which used age-stratified (< 75 years and ≥ 75 years [24, 26]) targets, and three set an individual BNP target (BNP level at discharge, reduction of 50% from baseline [22, 25, 31]). Algorithms for modifying treatment in the BNP-guided therapy groups differed slightly between RCTs, but all were based on stepwise titration of therapy according to clinical guidelines. Treatment for symptom-guided therapy groups used an algorithm designed to achieve a target HF score based on signs and symptoms (e.g. Framingham HF score and NYHA class) in five RCTs [20, 26, 28, 29, 31] and was entirely at the clinician’s discretion in seven RCTs [11, 21–25, 30].

**Table 1:** Characteristics of included studies and studies eligible for inclusion

Study	Country	Study period	Setting	Duration of follow-up	Follow-up schedule	BNP/NT-proBNP target	Clinical target	Primary endpoint	Treatment algorithm
Studies that provided IPD									
Anguita [20]	Spain	2006–2008	HF clinic	18 months	1, 2, 3, 6, 12, and 18 months	BNP level < 100 pg/ml	Framingham HF score of < 2	Composite of all-cause mortality and cardiovascular hospital admission	BNP group: therapy intensified to achieve target BNP Control group: therapy intensified to achieve target congestion score.
Northstar [27]	Denmark	2005–2009	HF clinic	2.5 years	Every 1–3 months at the discretion of the investigator	No set target	Clinical assessment	Composite of all-cause mortality and cardiovascular hospital admission	BNP group: checklist to evaluate need for further investigation or intensification of therapy when NT-BNP was > 30% from randomisation visit Control group: therapy evaluated and intensified at clinician discretion
Shochat [32] Published as abstract only	Israel	2007–2010	HF clinic	Median 11 months (IQR 3–22)	Every 1–2 months	No set target	Not known	All-cause mortality	BNP group: therapy intensified if NT-BNP was higher by > 30% from previous clinic visit Control group: not stated in abstract
Starbrite [31]	USA	2003–2005	HF clinic	4 months	Week 1 and then 1, 2, 3, and 4 months	Individual BNP at discharge	Individual congestion score	Composite of 90-day survival and hospital-free survival	BNP group: therapy intensified if BNP levels were 2 times greater than or less than the target BNP Control group: therapy intensified to achieve target congestion score
Upstep [24]	Sweden and Norway	2006–2009	HF clinic	≥ 12 months	Weeks 2, 6, 10, 16, 24, 36, 48, and then every 6 months	< 75 years: BNP level < 150 pg/ml ≥ 75 years: BNP level < 300 pg/ml	Clinical assessment	Composite of all-cause mortality, hospitalisation and worsening HF	BNP group: therapy intensified according to stepwise algorithm to achieve maximally tolerated or guideline recommended target doses Control group: therapy intensified at clinician discretion
Studies that provided aggregate data [33]									
Christchurch Pilot [29]	New Zealand	1998–1999	HF clinic	9.5 months	Every 3 months unless treatment targets not met	NT-proBNP level < 1700 pg/ml	Framingham HF score of < 2	Total cardiovascular events (mortality, hospital admission, new HF-related outpatient episode)	BNP group: therapy intensified according to stepwise algorithm to achieve target NT-BNP Control group: therapy intensified according to stepwise algorithm to achieve target HF score
Time-CHF^*^[26, 33]	Switzerland and Germany	2003–2006	HF clinic	18 months	1, 3, 6, 12, and 18 months	NT-proBNP less than 2× upper limit of normal: (< 400 pg/ml for patients < 75 yrs.; < 800 pg/ml for patients > = 75 years)	NYHA ≤II	Hospital-free survival	BNP group: therapy intensified according to stepwise algorithm to achieve target NT-BNP Control group: therapy intensified according to stepwise algorithm to achieve NYHA ≤II
Berger [21]	Austria	2003–2004	HF clinic	15 months	2 weekly, then 1, 3, 6, and 12 months	NT-proBNP < 2200 pg/L	Clinical assessment	Composite of all-cause mortality and HF re-hospitalisation	BNP group: therapy intensified according to set protocol to maintain target NT-BNP Control group: therapy intensified at clinician discretion
Prima [22]	Netherlands	2004–2007	HF clinic	24 months	2 weeks, 1 month, then 3 monthly for 2 years	Individual NT-proBNP level (lowest level at discharge or at 2 weeks follow-up)	Clinical assessment	Survival and hospital-free survival	BNP group: therapy intensified according to clinical guidelines to maintain target NT-BNP Control group: therapy intensified at clinician discretion
Signal-HF [25]	Sweden	2006–2009	Primary care	9 months	1, 3, 6, and 9 months	Individual NT-proBNP level (reduction of 50% from baseline)	Clinical assessment	Composite of survival, hospital-free survival and symptoms score	BNP group: stepwise algorithm to increase therapy to achieve target NT-BNP Control group: therapy intensified at clinician discretion
Battlescarred [28]	New Zealand	2001–2006	HF clinic	3 years	2 weekly until treatment target met then 3 monthly	NT-proBNP < 1300 pg/ml	Framingham HF score of < 2	All-cause mortality	BNP group: therapy intensified according to stepwise algorithm to achieve target NT-BNP and congestion score < 2 Control group: therapy intensified to achieve target congestion score < 2
Stars-BNP [23]	France	Not stated	HF clinic	15 months	Months 1, 2, and 3, and then 3 monthly thereafter	BNP level < 100 pg/ml	Clinical assessment	Composite of HF mortality or HF hospitalisation	BNP group: therapy intensified according to clinical guidelines to maintain target NT-BNP Control group: therapy intensified at clinician discretion
Protect [30]	USA	2006–2010	HF clinic	At least 6 months	As required to meet treatment target and then 3 monthly (for max 12 months)	NT-proBNP ≤ 1000 pg/ml	Clinical assessment	Composite of worsening HF, HF hospitalisation and cardiovascular events	BNP group: therapy intensified according to clinical guidelines to maintain target NT-BNP Control group: therapy intensified at clinician discretion
Guide-IT [11]	USA	2013–2016	HF clinic	15 months	Initial visits at 2 and 6 weeks and then every 3 months. A follow-up visit 2 weeks after any therapy adjustment	NT-proBNP < 1000 pg/ml	Clinical assessment	Composite of cardiovascular death and HF hospitalisation	BNP group: therapy intensified at clinician discretion but in line with clinical guidelines to achieve target NT-BNP Control group: therapy intensified at clinician discretion but in line with clinical guidelines
Eligible studies that did not provide IPD or aggregate data									
Karavidas [45] Published as abstract only	Greece	Not stated	Not stated	12 months	Not stated	Not stated but likely no set target	Clinical assessment	Not clear. Composite of all-cause mortality cardiovascular hospitalisation?	Not stated
Home [46] Clinical trial registration only	Ireland, UK, Australia and Canada	2011–2014	Not stated	6 months	1, 3, and 6 months	Not stated but likely no set target	Not stated	Average number of ‘hard’ events per subject (HF mortality, hospitalisation for HF, unplanned outpatient episodes for decompensated HF (including change in diuretic therapy)	BNP group: therapy intensified at clinician discretion using BNP information Control group: As above but without the BNP information
Optima [47] Published as poster only	Czech Republic	Not stated	Not stated	Not stated	Not stated	Not stated but likely a BNP lowering strategy	Clinical assessment	Composite of cardiovascular mortality, HF hospitalisation and outpatient episodes of worsening HF requiring an increase in diuretic by at least 50%	BNP group: therapy intensified to ‘normalise’ plasma BNP levels. Control group: therapy intensified at clinician discretion according to guidelines.
Koshkina et al. [48] Published as abstract only	Russian Federation	Not stated	HF clinic	Mean (SD) 10 ± 2.5 months	Not stated	NT-proBNP < 1000 pg/ml or at least 50% of the initial	Clinical assessment	Total cardiovascular events	Not stated
Ex Improve CHF [49] Study ongoing	Canada	2007–ongoing	HF clinic	Minimum 12 months	Not stated	No set target	Clinical assessment	Composite of all-cause mortality and HF hospitalisation	BNP group: therapy intensified at clinician discretion using BNP information Control group: As above but without the BNP information

^*^Time-CHF reported results separately for patients with heart failure with reduced ejection fraction (HFrEF) [26] and patients with heart failure with preserved ejection fraction (HFpEF) [33]

In the IPD dataset, the mean age of participants was 70 years, three quarters of patients were men, most patients had LV systolic dysfunction (median LVEF, 30%) and over 80%) had NYHA class II or III (Table 2). The patients in RCTs providing aggregate data had similar characteristics (Table 3).

**Table 2:** Baseline characteristics of patients in included studies

Study	Number of patients (BNP-guided/symptom-guided)	Age (years) Mean (SD)	Patients ≥ 75 years *n* (%)	Male *n* (%)	LVEF (%) Median (IQR)^*^	LVEF ≥ 40%^**^n (%)	NYHA class I/II/III/IV	Smoking status (non-smoker/ex-smoker/current smoker)	Body mass index, kg/m^2^(mean, SD)	Systolic BP, mmHG (mean, SD)	Diastolic BP, mmHG (mean, SD)
Studies that provided IPD											
Anguita [20]	60 (30/30)	69 (10)	18/54 (33%)	41/60 (68%)	40 (26, 65)	27/55 (49%)	3/38/19/0	37/7/16	–	–	–
Northstar [27]	407 (199/208)	73 (8)	186/407 (46%)	309/407 (76%)	30 (25, 35)	36/402 (9%)	80/268/59/0	47/114/110	26 (5)	127 (19)	73 (12)
Shochat [32]	120 (60/60)	70 (11)	50/120 (42%)	103/120 (86%)	30 (25, 35)	11/75 (15%)	1/55/41/16	34/0/40	–	125 (21)	74 (11)
Starbrite [31]	130 (65/65)	60 (15)	24/130 (18%)	91/130 (70%)	20 (15, 25)	0/129 (0%)	–	–	29 (8)	111 (21)	69 (13)
Upstep [24]	268 (140/128)	71 (10)	105/268 (39%)	196/268 (73%)	–	0/268 (0%)	0/83/147/37	–	27 (5)	–	–
All	985 (494/491)	70 (11)	383/979 (39%)	740/985 (75%)	30 (20, 35)	74/929 (8%)	84/444/266/53	118/121/166	27 (5)	124 (21)	73 (12)
Studies that provided aggregate data^§^											
Christchurch Pilot [29]	69 (33/36)	70 (10)	24/69 (35%)	53/69 (7%)	27 (8)	0/69 (0%)	~ 70% (class II)	–	–	127 (SD not provided)	76 (SD not provided)
Time-CHF HFrEF [26]	499 (251/248)	76 (8)	289/499 (58%)	327/499 (66%)	30 (8)	0/499 (0%)	371/499 (74%) (≥ class III)	–	25 (4)	119 (19)	–
Time-CHF HFpEF [33]	123 (59/64)	80 (7)	–	42/123 (34%)	56 (6)	123/123 (100%)	0/21/82/20	–	27 (5)	136 (23)	74 (12)
Berger [21]	188 (92/96)	71 (12)	88/188 (47%)	147/188 (78%)	29 (9)	11/188 (6%)	All patients class III–IV	–	–	121 (18)	72 (12)
Prima [22]	345 (174/171)	72 (12)	166/345 (48%)	197/345 (57%)	36 (14)	93/345 (27%)	37/234/74	166/105/74	–	118 (21)	69 (11)
Signal-HF [25]	252 (127/125)	78 (7)	184/252 (73%)	180/252 (71%)	32 (8)	5/252 (2%)	0/154/96/0	–	–	134 (22)	74 (12)
Battlescarred [28]	242 (121/121)	74 (9)	138/242 (57%)	157/242 (65%)	39 (15)	90/242 (37%)	24/162/52/4	–	–	124 (23)	71 (13)
Stars-BNP [23]	220 (110/110)	66 (5)	–	127/220 (58%)	31 (8)	–	–	101/220 (current smokers)	–	–	–
Protect [30]	151 (75/76)	63 (14)	38/151 (25%)	127/151 (84%)	27 (9)	0/151 (0%)	129/151 (85%) (class II–III)	92/48/11	29 (6)	110 (16)	66 (9)
Guide-IT [11]	894 (446/448)	62 (51 to 70) in BNP-guided group, 64 (54–72) in symptom-guided group^***^	161/894 (18%)	608/894 (68%)	25 (19 to 30)^***^	All had LVEF ≤ 40%	59/447/358/17 of 881	–	–	114 (102 to 128) in BNP-guided group, 114 (101 to 128) in symptom-guided group^***^	–

^*^Mean (SD) for studies providing aggregate data. ^**^≥ 45% for studies providing aggregate data. ^***^Median and interquartile range, ^§^ data from original reports or IPD meta-analysis by Troughton et al. [9] and Brunner La-Rocca et al. [17]. Missing data: Age, Anguita—6 patients with missing data; LVEF, Anguita—5 patients with missing data; Northstar—5 patients with missing data; Shochat—45 patients with missing data; Starbrite—1 patient with missing data; BMI, Northstar—4 patients with missing data; Starbrite—45 patients with missing data; Upstep—3 patients with missing data; SBP, Shochat—37 patients with missing data; Starbrite—1 patient with missing data; DBP, Northstar—1 patient with missing data; Shochat—37 patients with missing data; Starbrite—1 patient with missing data

**Table 3:** BNP/NT-proBNP (pg/ml) levels at baseline and end of follow-up in the BNP-guided therapy group and symptom-guided therapy group

BNP/NT-proBNP (pg/ml)^*^														
Studies that provided IPD						Studies that provided aggregate data								
	Anguita [20]	Northstar [27]	Shochat [32]	Starbrite [31]	Upstep [34]	Christchurch Pilot [29]	Time-CHF [26, 33]	Berger [21]	Prima [22]	Signal-HF [25]	Battlescarred [28]	Stars-BNP [23]	Protect [30]	Guide-It [11]
	*n* = 60	*n* = 407	*n* = 120	*n* = 130	*n* = 268	*n* = 69	*n* = 499	*n* = 278	*n* = 345	*n* = 252	*n* = 364	*n* = 220	*n* = 151	
BNP guided-therapy:														
Baseline^*^	34 (7, 83)	1884 (1385, 2955)	1905 (1099, 4488)	453 (221, 1135)	601 (346, 946)	1839	HFrEF 3998 (2075, 7220) HFpEF 2210 (1514–4081)	2216 (355, 9649)	2961 (discharge) (1383, 5144)	2661 (2.1)	2012 (516, 10,233)	352 (260)	2344	2568
End of follow-up	8 (3, 83)	–	1765 (476, 3966)	413 (111, 894)	–	1169	–	–	2529	2360	1610 (6 months)	284 (180) (3 months)	1125	1209
Difference	2 (−31, 28)	–	−81 (− 1273, 512)	−14 (− 461, 248)	–	− 670	–	–	− 432 (− 1392, 297)	−301	−402	− 68	1219	1359
% change from baseline:	6%	–	−4%	−3%	–	−36%	–	–	−15%	− 11%	− 20%	− 19%	−52%	− 53%
Symptom-guided therapy:														
Baseline^*^	22 (5, 104)	2042 (1390, 3560)	1569 (784, 4919)	441 (189, 981)	609 (376, 952)	2127	HFrEF 4657 (2455, 7520) HFpEF 2191 (1478, 4890)	2469 (355, 18,487)	2936 (discharge) (1291, 5525)	2429 (2.1)	1996 (425, 6588)	–	1946	2678
End of follow-up	39 (6, 104)	–	1822 (618, 4489)	471 (236, 1180)	–	2102	–	–	2364	2067	1537 (6 months)	–	1844	1397
Difference	4 (−20, 46)	–	73 (− 554, 1245)	51 (− 130, 288)	–	−25	–	–	− 572 (− 1329, 434)	− 362	− 459	–	102	1281
% change from baseline:	18%	–	5%	12%	–	−1%	–	–	−19.5%	− 15%	−23%	–	−5%	− 48%

^*^Median (IQR) reported for all studies except Christchurch Pilot (type of summary statistic not reported), Protect (median only reported), Signal-HF (geometric mean reported), and Stars-BNP (mean reported)

For all studies, BNP values at discharge from randomisation visit were used (assumed values at discharge reported if not stated otherwise in trial reports)

For Northstar and Upstep, BNP values at the end of follow-up were not available. For the remaining studies providing IPD, the difference between the baseline and end of follow-up was calculated as the median (IQR) change from baseline across patients. For studies providing aggregate data, the change from baseline was calculated by taking the average BNP at end of follow-up from the average BNP at baseline.

For all studies, the % change from baseline is calculated as the average difference as a percentage of the average baseline BNP

Eleven out of the 14 included RCTs (79%) were rated as having a high risk of bias across at least one risk domain (Additional file 2: Appendix 2). The main factor that contributed to ratings of high risk of bias was the lack of blinding (of participants and care-giving clinicians). None of the funnel plots generated for the outcomes with more than 10 studies contributing data suggested marked asymmetry (Additional file 3: Appendix 3). There were no significant issues identified with the IPD datasets provided. The overall quality of the body of evidence for all outcomes varied from low to very low (Table 4).

**Table 4:** Summary of findings table

Serial B-type natriuretic peptide (BNP) blood testing to guide up-titration of medication compared to symptom-guided up-titration of medication in patients with heart failure (HF)						
Patient or population: patients with heart failure (HF) Setting: secondary care Intervention: serial B-type natriuretic peptide (BNP) blood testing to guide up-titration of medication Comparison: symptom-guided up-titration of medication						
Outcome No. of participants (studies)	Relative effect (95% CI)	Anticipated absolute effects (95% CI)			Certainty	What happens
		Without serial B-type natriuretic peptide (BNP) blood testing to guide up-titration of medication	With serial B-type natriuretic peptide (BNP) blood testing to guide up-titration of medication	Difference		
All-cause mortality follow up: range 3 to 30 months No. of participants: 3691 (13 RCTs)	HR 0.87 (0.71 to 1.01)	19.9%	17.5% (14.6 to 20.1)	2.3% fewer (5.3 fewer to 0.2 more)	⨁⨁◯◯ Low^a,b^	BNP-guided therapy may result in little to no difference in all-cause mortality.
Death related to HF follow up: range 12 to 15 months No. of participants: 488 (2 RCTs)	Two studies reported death related to HF. There were no significant differences between the BNP-guided therapy and the symptom-guided therapy groups in either study (3/110 vs. 9/110, respectively, and 21/140 vs. 16/128, respectively).				⨁◯◯◯Very low ^a,b,c^	It is uncertain whether BNP-guided therapy prevents death related to HF because the quality of the evidence is very low.
Cardiovascular death follow up: range 9 to 23 months No. of participants: 1909 (5 RCTs)	OR 0.88 (0.67 to 1.16)	13.7%	12.3% (9.6 to 15.6)	1.4% fewer (4.1 fewer to 1.9 more)	⨁⨁◯◯ Low ^a,b^	BNP-guided therapy may lead to little or no difference in cardiovascular death.
All-cause hospitalisation follow up: range 3 to 30 months No. of participants: 984 (7 RCTs)	HR 0.97 (0.85 to 1.10)	57.2%	56.1% (51.4 to 60.7)	1.1% fewer (5.8 fewer to 3.5 more)	⨁⨁◯◯ Low^a,b^	BNP-guided therapy may result in little or no difference in all-cause hospitalisation.
HF hospitalisation follow up: range 9 to 30 months No. of participants: 2655 (8 RCTs)	HR 0.81 (0.68 to 0.98)	34.1%	28.6% (24.7 to 33.5)	5.4% fewer (9.4 fewer to 0.6 fewer)	⨁◯◯◯ Very low^a,b,d^	It is uncertain whether BNP-guided therapy reduces hospital admissions for HF because the quality of evidence is very low.
Adverse events follow up: range 9 to 18 months No. of participants: 2055 (5 RCTs)	OR 1.29 (1.04 to 1.60)	24.4%	29.4% (25.1 to 34.1)	5.0% more (0.7 more to 9.7 more)	⨁⨁◯◯ Low^a,b^	BNP-guided therapy may lead to an increase in adverse events.
Quality of life follow up: range 10 to 30 months No. of participants: 1884 (6 RCTs)	Six studies reported data on QoL (five used the Minnesota Living with Heart Failure Questionnaire and one used SF-36) in their published report. Data could not be combined in a meta-analysis because changes in QoL were reported differently in each study. Only one study reported a significant improvement in QoL in the BNP-guided therapy group vs. symptom guided therapy group; five reported no difference between groups. Three additional studies included a statement in their published report saying that that there was no difference in QoL between groups and one included a statement saying that results of QoL analyses were not reported in the manuscript.				⨁◯◯◯ Very low^a,b,c^	It is uncertain whether BNP-guided therapy improves quality of life because the quality of the evidence is very low.

*The risk in the intervention group (and its 95% confidence interval) is based on the assumed risk in the comparison group and the relative effect of the intervention (and its 95% CI)

*CI* confidence interval; *HR* hazard ratio; *OR* odds ratio

GRADE Working Group grades of evidence

High certainty: we are very confident that the true effect lies close to that of the estimate of the effect

Moderate certainty: we are moderately confident in the effect estimate; the true effect is likely to be close to the estimate of the effect, but there is a possibility that it is substantially different

Low certainty: our confidence in the effect estimate is limited; the true effect may be substantially different from the estimate of the effect

Very low certainty: we have very little confidence in the effect estimate; the true effect is likely to be substantially different from the estimate of effect

^a^None of the RCTs were blinded (either patients or healthcare professionals) so at risk of bias due to deviations from intended interventions (performance bias)

^b^Most RCTs did not include patients with HF with preserved ejection fraction (HFpEF). Women and patients > = 75 years were also under-represented

^c^Narrative synthesis was conducted; estimates are not precise

^d^*I*^2^ statistic (46%) suggests moderate heterogeneity

### Primary outcome

**Fig. 2:**
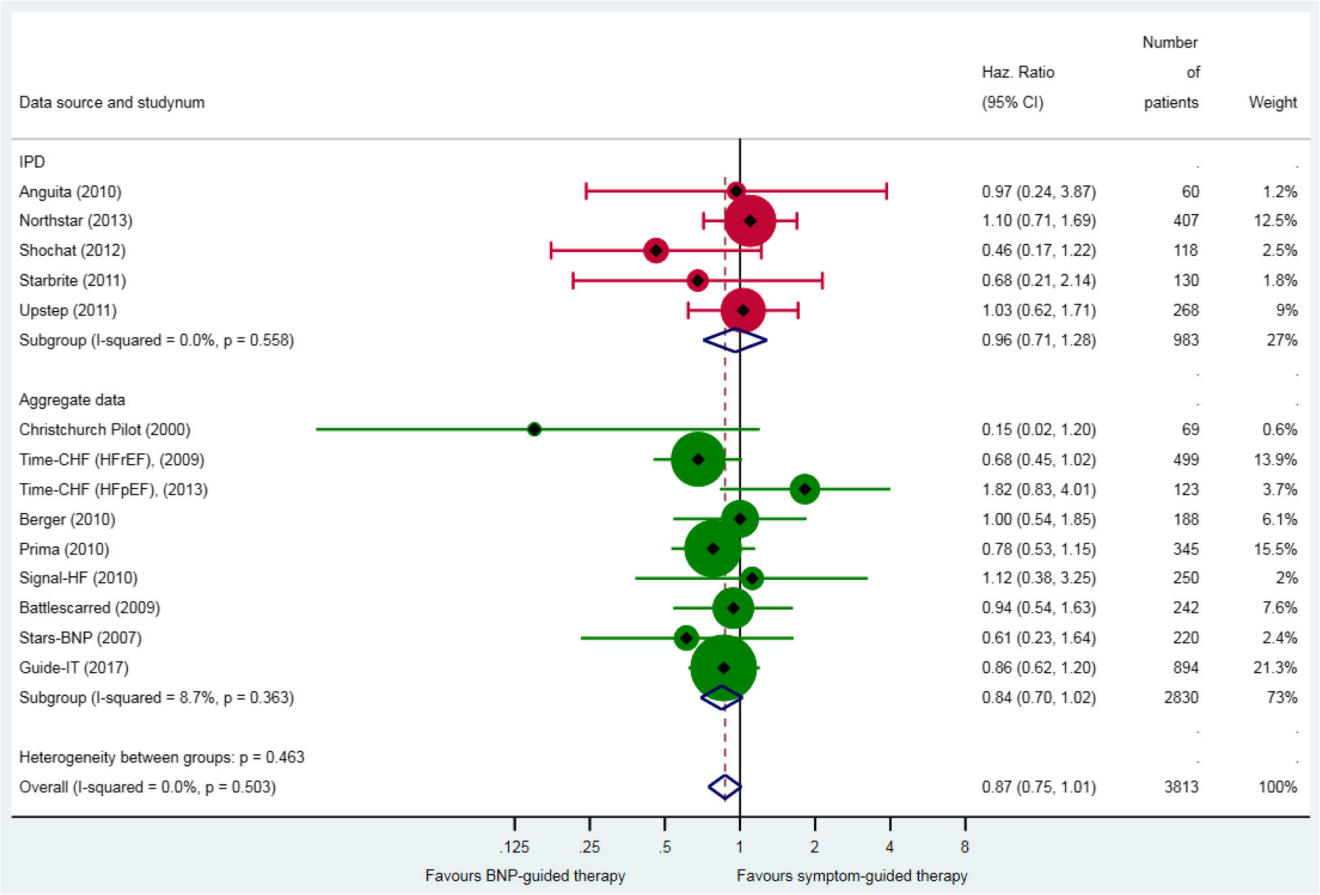
All-cause mortality. Unadjusted individual hazards ratios (HR) with 95% confidence intervals (CI) presented within IPD, aggregate data, and overall. Time-CHF reported results separately for patients with heart failure with reduced ejection fraction (HFrEF) [26] and patients with heart failure with preserved ejection fraction (HFpEF) [33]. HR for all-cause mortality was not available for the Protect study [30]. The HR and 95% CI from Guide-It [11] was adjusted for age, sex, left ventricular ejection fraction, NT-proBNP, and the presence of diabetes mellitus. Note: weights are from random effect analysis

Across 13 RCTs that reported all-cause mortality, 17% (320/1845) of patients in the BNP-guided therapy group, and 20% (367/1846) of patients in the symptom-guided therapy group died during follow up. Median follow-up in the five RCTs that provided IPD was 18 months (IQR 8–27). BNP-guided therapy did not reduce the hazard of death from any cause compared with symptom-guided therapy (HR 0.87, 95% CI 0.75 to 1.01) (Fig. 2). There was no significant heterogeneity between RCTs. The sensitivity analysis excluding the two RCTs that did not use a BNP-lowering strategy did not alter this finding (HR 0.86, 95% CI 0.73 to 1.01). The sensitivity analysis combining the effect estimates from three RCTs that were judged to have had good allocation concealment showed no difference in the hazard of death between groups (HR 0.93, 95% CI 0.60–1.44).

### Secondary outcomes

**Fig. 3:**
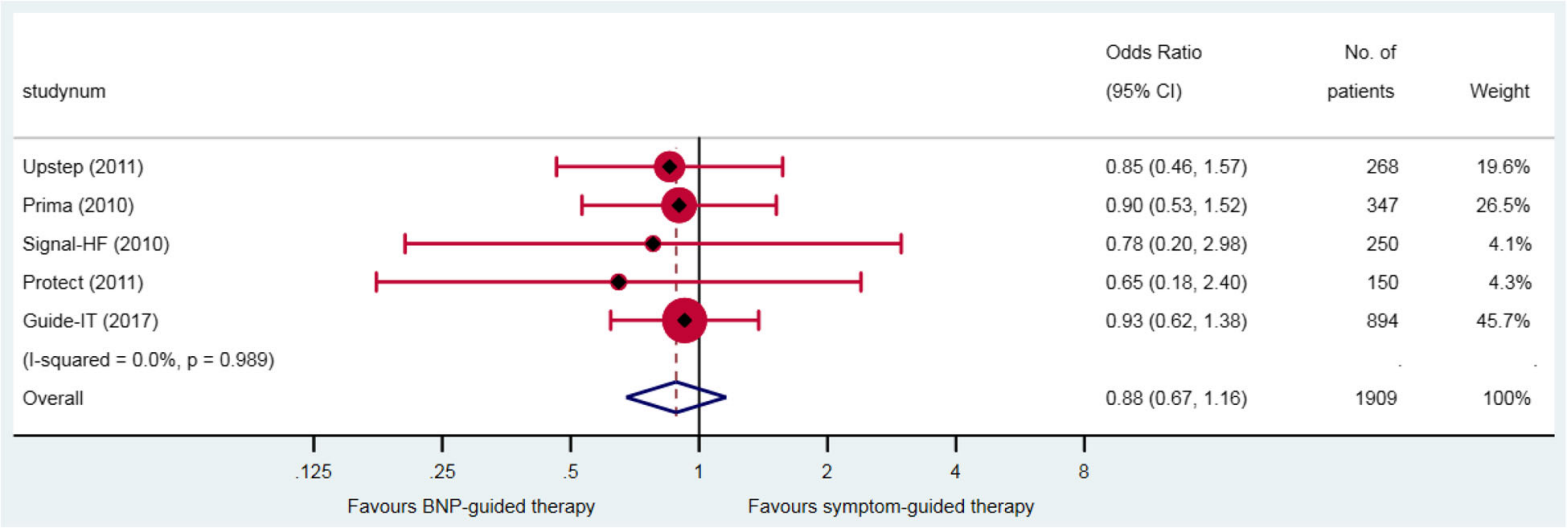
Cardiovascular mortality. Odds ratio (OR) with 95% confidence intervals (CI) for five aggregate data studies. Note: weights are from random effect analysis

Five RCTs provided aggregate data on numbers of patients with cardiovascular death; these showed that 12% (120/963) of patients in the BNP-guided therapy group and 14% (130/946) of patients in the symptom-guided therapy group died because of a cardiovascular cause. BNP-guided therapy did not reduce the odds of cardiovascular death (OR 0.88, 95% CI 0.66–1.16) (Fig. 3). Only two studies provided aggregate data on death due to HF; Stars-BNP [23] and Upstep [24] showed that 3% (3/110) and 15% (21/140), respectively, of patients in the BNP-guided therapy group, and 8% (9/110) and 12.5% (16/128), respectively, of patients in the symptom-guided therapy group had a death directly attributable to HF.

**Fig. 4:**
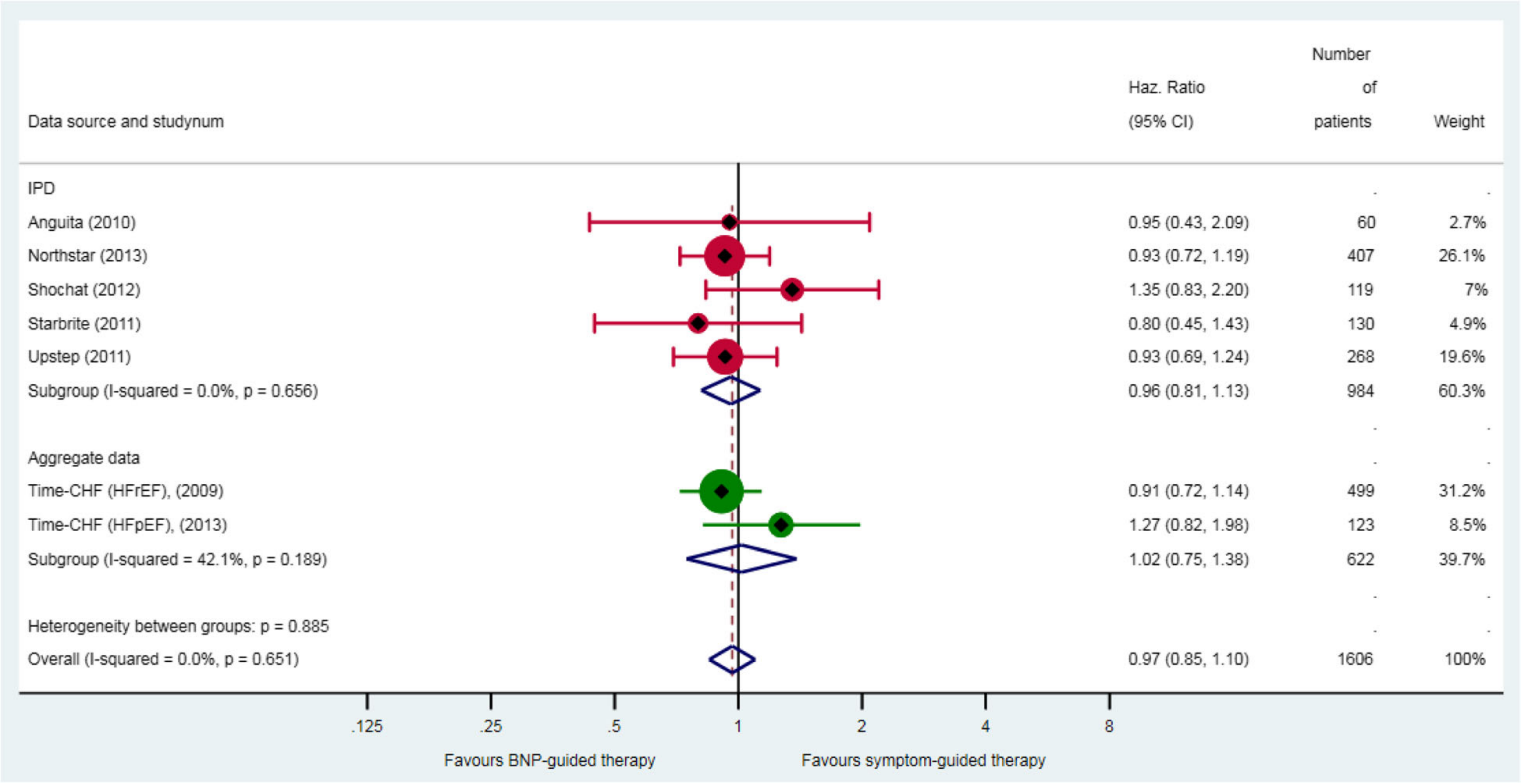
All-cause hospitalisation. Unadjusted individual hazards ratios (HR) with 95% confidence intervals (CI) presented within IPD, aggregate data, and overall. HR for all-cause hospitalisation was only available for Time-CHF (HFrEF and HFpEF [33]). Note: weights are from random effect analysis.

Across six RCTs with data on all-cause hospitalisation, 58% (285/493) of patients in the BNP-guided therapy group had at least one hospital admission, compared with 57% (281/491) of patients in the symptom-guided therapy group. BNP-guided therapy did not reduce the hazard of all-cause hospitalisation (HR 0.97, 95% CI 0.85–1.10) (Fig. 4). The results did not differ in the analysis restricted to RCTs that used a BNP-lowering strategy (HR 0.95, 95% CI 0.81–1.11).

**Fig. 5:**
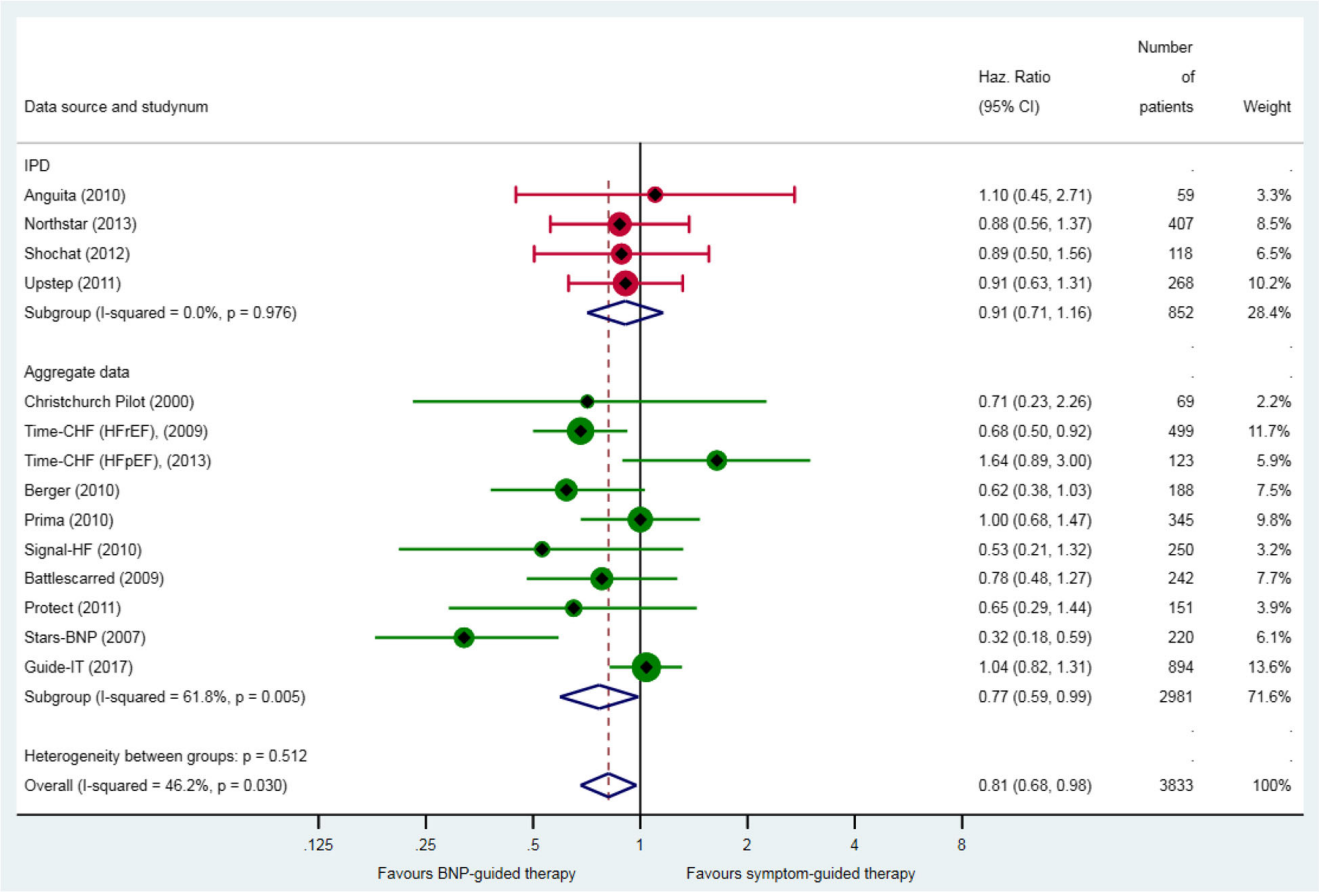
Heart failure (HF) hospitalisation. Unadjusted individual hazards ratios (HR) with 95% confidence intervals (CI) presented within IPD, aggregate data, and overall. Note: weights are from random effect analysis

Across eight RCTs that provided data on numbers of patients with HF hospitalisation, there were 392/1328 patients (29.5%) who had at least one hospitalisation for HF in the BNP-guided therapy group, compared with 452/1327 patients (34%) in the symptom-guided therapy group. BNP-guided therapy led to a lower hazard of hospitalisation due to HF (HR 0.81, 95% CI 0.68–0.98) (Fig. 5**)**. The results did not differ in the sensitivity analysis restricted to RCTs that set a BNP target (HR 0.77, 95% CI 0.64–0.99). The sensitivity analysis with respect to allocation concealment was not performed because only two RCTs were classified as having a low risk of bias. In all meta-analyses (for primary and secondary outcomes), the results from the fixed-effects meta-analyses did not differ from the random-effects meta-analyses results.

### Subgroup analyses

Stratum-specific treatment effects are reported in Additional file 4: Appendix 4. For all-cause mortality, there was a significant interaction between treatment strategy and age (*p* = 0.034, 11 RCTs), and treatment strategy and LVEF (*p* = 0.026, 10 RCTs). BNP-guided therapy was beneficial for trial participants < 75 years old (HR 0.70, 95% CI 0.53–0.92) but not for trial participants ≥ 75 years old (HR 1.07, 95% CI 0.84–1.37). Similarly, BNP-guided therapy was beneficial for trial participants with HFrEF (HR 0.84, 95% CI 0.71–0.99), but not those with HFpEF (HR 1.33, 95% CI 0.83–2.11). This effect was largely driven by one RCT (Time-CHF); excluding this from the analysis attenuated the protective effect in the lower LVEF subgroup was attenuated (HR 0.89, 95% CI 0.73–1.06).

There were no significant interactions between treatment strategy and any of the other covariates investigated in the subgroup analyses for any of the outcomes (*p* > 0.05). However, for age and LVEF, stratum-specific estimates for the secondary outcomes (all-cause and HF hospitalisations) were consistent with those for all-cause mortality, suggesting a protective effect of BNP-guided therapy.

### Changes in BNP from baseline to end of follow-up

**Fig. 6:**
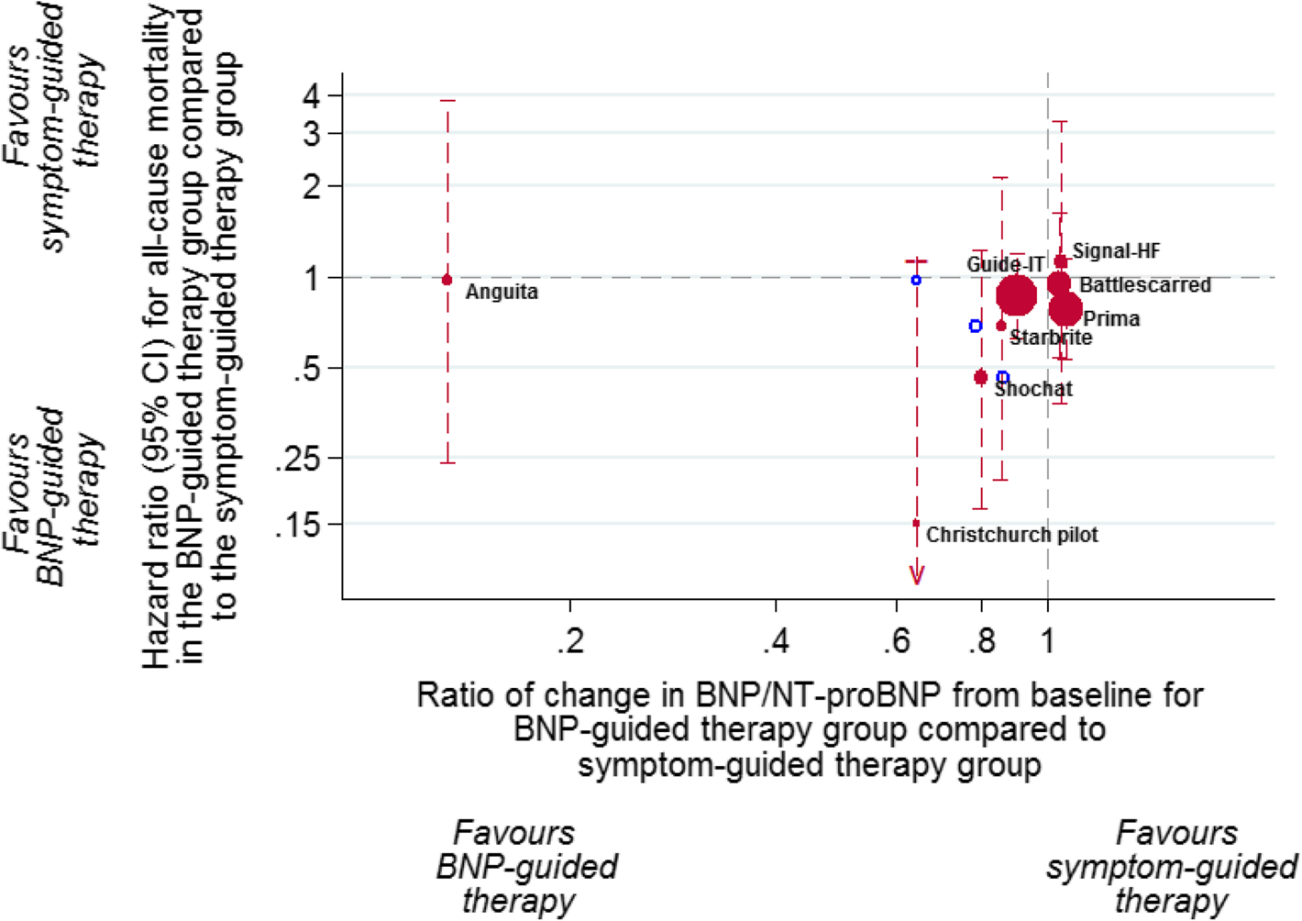
Relationship between hazard ratios (HR) for all-cause mortality and the ratio of change in BNP/NT-proBNP from baseline between the BNP-guided therapy group and symptom-guided therapy group. Filled circles represent the ratio of the change calculated using aggregate data, and open circles represent the ratio of the change calculated using IPD; the change in the position on the *x*-axis shows the differences between the two analyses methods, while the position on the *y*-axis remains the same

BNP levels at baseline and end of follow-up were available for nine RCTs (Table 3). In six of these [11, 22, 25, 28–30], BNP levels decreased in both the BNP-guided therapy group and the symptom-guided therapy group. There was no consistent relationship between the change in BNP from baseline between groups and the HR for all-cause mortality (Fig. 6). RCTs that provided evidence for a relationship (i.e. studies with the most extreme HRs for mortality favouring BNP guided-therapy and in which BNP fell substantially more in the BNP-guided group than in the symptom-guided group) provided least weight in the meta-analysis. Calculating the relative change between groups using IPD (for studies that provided IPD) provided even less evidence for a relationship.

### Medication changes

We could not combine IPD and aggregate data to investigate the association between changes in medication and outcomes because changes in medication were inconsistently reported in studies on both IPD studies and aggregate data studies.

### Adverse events and discontinuation

**Fig. 7:**
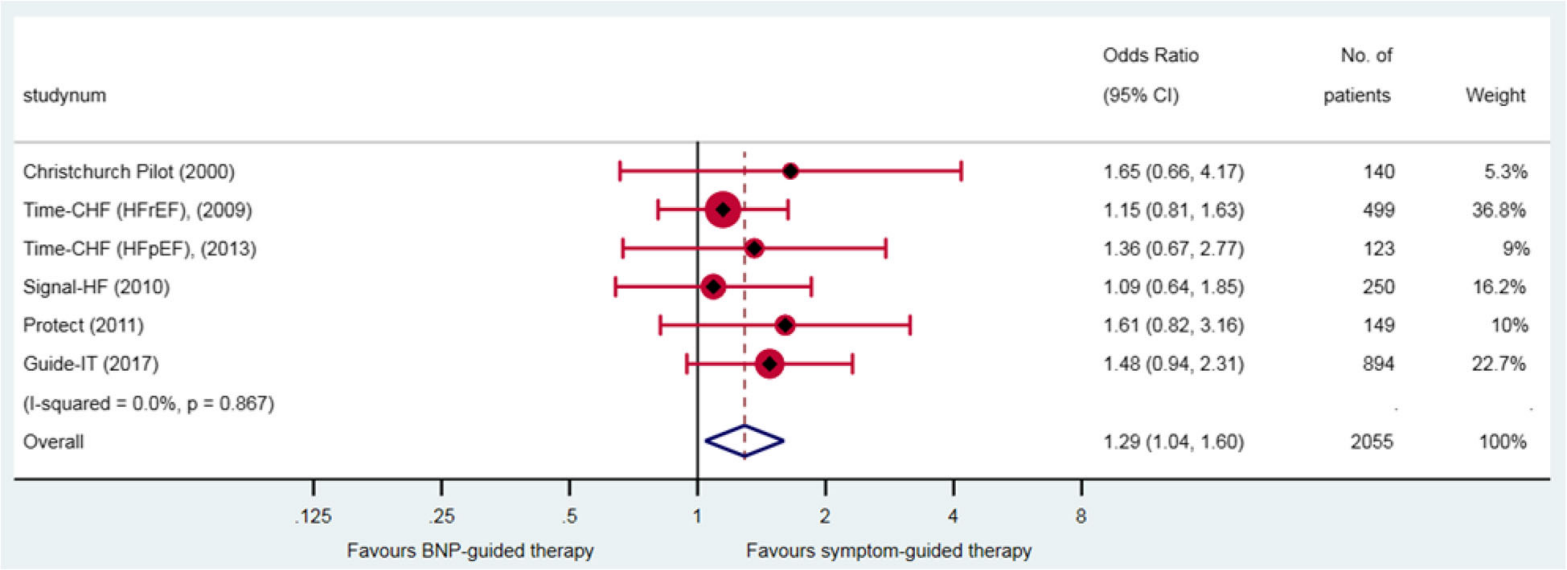
Total adverse events. Odds ratios (OR) and 95% confidence intervals (CI) for five aggregate data studies. Note: weights are from random effect analysis

None of the IPD studies provided data on adverse events. Five aggregate data studies [11, 25, 26, 29, 30, 33] provided data on total number of adverse events by group; these showed that 29% (293/1023) of patients in the BNP-guided therapy group and 14% (130/946) of patients in the symptom-guided therapy group experienced an adverse event. Adverse events were significantly more frequent in the BNP-guided therapy group compared with that in symptom-guided therapy group (OR 1.29, 95% CI 1.04 to 1.60) (Fig. 7). Adverse events most commonly reported included renal impairment and hypotension, one study reported additional adverse events such as hyper/hypokalaemia, anaemia, fever, dizziness, gastrointestinal bleeding, respiratory infection, and syncope.

### Quality of life (QoL)

None of the IPD studies provided QoL data both at baseline and follow-up. QoL data were available from aggregate data in six studies (including the published reports of two IPD studies: Northstar [27] and Upstep [24]). These could not be pooled in a meta-analysis because changes in QoL were reported differently in each study. Five studies assessed QoL using the Minnesota Living with Heart Failure questionnaire [22, 26–28, 30], and one study [24] assessed QoL using the SF-36 questionnaire. Northstar [27] reported no change in QoL in either group [median (IQR) change 0 (− 6 to 2) and 0 (− 5 to 6) between baseline and end of study visit (6 months to 4.5 years), in the BNP-guided therapy group and symptom-guided therapy group, respectively]. Three of the aggregate data studies showed that QoL improved significantly and similarly in both groups: Battlescarred [28]: mean (SD) 36.5 (22.7) and 36.6 (23.1) at baseline vs. 28.8 (21.6) and 26.5 (22.0) at 12 months in the BNP-guided therapy group and symptom-guided therapy group, respectively; Prima [22] (median (IQR), 47 (34 to 62) and 48 (36 to 60) at baseline vs. 20 (3–36) and 23 (10–38) at 12 months follow up, in the BNP-guided therapy group and symptom-guided therapy group, respectively; Time-CHF [26] (mean (SD), 38.3 (20.2) and 40.2 (20.3) at baseline vs. 27.7 (17.90 and 27.0 (18.6) at 12 months, in the BNP-guided therapy group and symptom-guided therapy group, respectively. Only one of the aggregate data studies showed a greater improvement in the BNP-guided therapy group compared with the symptom-guided therapy group [Protect [30] (median improvement between baseline and follow-up at 12 months, − 10.0 vs. − 5.0, *p* = 0.05, in the BNP-guided therapy group and symptom-guided therapy group, respectively]. The Upstep study [24] assessed quality of life using the SF-36 questionnaire (eight domains) and found no significant differences between groups [34]. The remaining studies did not provide data on QoL; three studies included a statement to say that the change/improvement in QoL was similar in both groups [20, 25, 29], one study did not mention quality of life [31], while the Guide-It study included a statement saying that the results of QoL analyses were not reported in their manuscript [11].

## Discussion

### Main findings

Our meta-analysis, including data for up to 3968 patients with HF (1982 randomised to BNP-guided therapy and 1986 randomised to symptom-guided therapy) suggests that BNP-guided therapy may result in little to no difference in all-cause or cardiovascular mortality. It is uncertain whether BNP-guided therapy reduces hospital admissions for HF because the quality of evidence is very low. BNP-guided therapy may lead to an increase in adverse events. A previous IPD meta-analysis by Troughton et al. [9] (which excluded the subgroup of participants with HFpEF from the Time-CHF RCT) showed a 18% reduction in the hazard of death from any cause (HR 0.82, 95% CI 0.67–1.00) and a 26% reduction in the hazard of hospital admission for HF (HR 0.74, 95% CI 0.60–0.90), but the authors did not assess the quality of evidence and therefore reached different conclusions. The results from our subgroup analyses showed more benefit of BNP-guided therapy in patients < 75 years old and patients with HFrEF, which is consistent with the analyses reported by Troughton et al. [9] and Brunner La-Rocca et al. [17].

### Was the treatment effect a result of decreasing BNP or up-titration of medication?

The observed benefit in the BNP-guided therapy group could not be attributed to changes in BNP levels during follow-up between groups (Fig. 5). There was no consistent relationship between the relative BNP change from baseline between groups and the hazard ratio for all-cause mortality. Although the smaller RCTs showed a relatively large BNP change between groups and lower hazard ratios, this was not reflected in the larger RCTs that provided most weight in the meta-analysis. The meta-analysis by Troughton et al. [9] showed that BNP levels decreased by 35% in the BNP-guided therapy group and 32% in the symptom-guided therapy group. Similarly, the Guide-IT RCT showed a 53% decrease in the BNP-guided therapy group and a 48% decrease in the symptom-guided therapy group.

Although we could not determine whether and how HF medication doses changed during follow up, the meta-analysis by Troughton et al. [9] showed no differences in medication dose changes between groups, except for a modest increase in doses of angiotensin-converting enzyme inhibitors (ACEi)/angiotensin II receptor blockers (ARB) in the BNP-guided therapy group (8.4% increase, 3.4 to 13, vs. − 1.2% decrease, − 6.1 to 3.7 in the symptom-guided therapy group). While treatment with ACEi and ARB according to guidelines has been shown to reduce the risk of death and hospitalisation in both RCTs and large registries [35–38]; over 89% of patients in the RCTs were already receiving these medications [9], so it is unclear whether the relatively small dose increases in the BNP-guided therapy group were responsible for the benefit observed. Furthermore, the Guide-IT RCT showed modest intensification of HF medications, including ACEi and ARB, in both groups.

### BNP-lowering vs. BNP-monitoring strategy

In our meta-analysis, we included all RCTs that used serial BNP measurements to guide HF therapy, regardless of the guiding strategy used. There were several reasons for this. First, we aimed to provide realistic treatment effect estimates given that, in the absence of established guidelines describing how treatment should be guided by BNP, clinicians are likely to use BNP levels to manage their patients in diverse ways (e.g. to check the status quo, to lower BNP as much as possible, or to a target). Second, the two strategies are not fundamentally dissimilar, since both will prompt a patient review with intensification of medications if considered appropriate. Third, RCTs evaluating BNP-lowering were themselves heterogeneous in design, treatment strategies (in both the BNP group and the control group), and BNP target. Finally, we wished to include all studies in order to avoid publication related biases, data availability bias, and reviewer selection bias [39–41]; previous meta-analyses did not publish a priori protocols. These biases can lead to meta-analyses being biased towards more favourable treatment effects [41, 42] and have been highlighted as a potential problem in meta-analyses that use IPD [41]. The exclusion of two studies that did not target a specific BNP level [27, 32] did not alter the findings of our meta-analysis.

### Strengths and limitations of this study

Our meta-analysis has several strengths. We systematically identified all RCTs evaluating BNP-guided therapy in HF patients, included all RCTs for which IPD or aggregate data were available, and conducted meta-analyses in accordance with a pre-specified protocol [12] and published guidelines. There was no evidence of publication bias or a small study effect (for all-cause mortality and HF hospitalisation, which had data from more than 10 studies).

The main limitation was our inability to obtain IPD from most of the RCTs included in the meta-analysis by Troughton et al. [9], which constrained our sub-group analyses. Other limitations arose from the design of the RCTs: heterogeneity in how BNP-guided therapy and symptom-guided therapy was administered; restricted eligibility (mainly younger patients with HFrEF and without co-morbidities), limiting the applicability of the results to the broader HF population; and the potential for bias because most RCTs did not blind clinicians or patients to treatment allocation. This lack of blinding means that co-interventions affecting outcomes could have been initiated by either the doctors or the participants themselves, conditional on their knowledge of the allocation. Also, despite combining results from 14 RCTs, the pooled sample size (up to 3968 patients with HF) was relatively small in comparison with sample sizes in other meta-analyses in this patient population (some of which included over 13,000 patients [43]); therefore, chance may explain some of the apparently significant findings. Finally, data on adverse events were not reported consistently and therefore only five studies contributed data for a meta-analysis.

### Implications for clinicians and policy-makers

Our meta-analysis has shown that BNP-guided treatment in hospital cardiology clinics significantly reduced HF hospitalisation but not all-cause or cardiovascular mortality. However, this conclusion may not be applicable to other health settings and HF patients who were not eligible (older patients with HFpEF). By contrast, across many European countries, cardiologists do not lead the management of patients with HF, and about half of all patients have HFpEF. Patients with HFpEF tend to be older with more comorbidities than their HFrEF counterparts. There are significant gaps and variation in the medical care of HF patients, and there is evidence that not all patients are receiving optimal treatment according to guidelines [44]. It therefore appears more prudent in the first instance to ensure adherence to guidelines for managing HF before recommending BNP-guided therapy.

## Conclusion

The conclusions about the efficacy of BNP-guided therapy are uncertain because the findings are of borderline statistical significance and the overall quality of the evidence varied from low to very low. We could not identify an optimal BNP monitoring strategy and no group of researchers has defined one. Therefore, consensus about an optimal BNP monitoring strategy should urgently be sought, preferably through a formal process involving cardiologists, general practitioners, and patients. It is striking that BNP levels decreased, and HF medications increased in both the BNP-guided therapy and symptom-guided therapy groups in the RCTs; this strongly suggests that HF management outside the RCTs was suboptimal. The reasons why not all patients receive care according to guidelines is unclear; understanding why may require qualitative research with different types of practitioner who care for HF patients.

## Additional files

Additional file 1: Appendix 1. Literature search. (DOCX 18 kb)

Additional file 2: Appendix 2. Risk of bias. (DOCX 132 kb)

Additional file 3: Appendix 3. Funnel plots. (DOCX 41 kb)

Additional file 4: Appendix 4. Subgroup analyses. (DOCX 851 kb)

## Abbreviations

ACEi: Angiotensin-converting enzyme inhibitors
ARB: Angiotensin II receptor blockers
BNP: B-type natriuretic peptide
CI: Confidence interval
HF: Heart failure
HFpEF: Heart failure with preserved ejection fraction
HFrEF: Heart failure with reduced ejection fraction
HR: Hazard ratio
IPD: Individual participant data
LV: Left ventricular
LVEF: Left ventricular ejection fraction
NT-proBNP: N-terminal pro-B-type natriuretic peptide
NYHA: New York Heart Association
QoL: Quality of life
RCT: Randomised controlled trial
