# Supplementary material for: B-type natriuretic peptide-guided therapy for heart failure (HF): a systematic review and meta-analysis of individual participant data (IPD) and aggregate data
Source: Syst Rev. 2018 Jul 31;7:112. doi: 10.1186/s13643-018-0776-8 (PMC6069819; doi:10.1186/s13643-018-0776-8)
Supplement: Supplementary file 1 — Appendix 1. Literature search. (DOCX 18 kb) [file 13643_2018_776_MOESM1_ESM.docx]

Appendix 1

Literature search

**OVID-MEDLINE, MEDLINE-IN-PROCESS**

1. (BNP adj5 (guide$ or monitor$ or target$ or predict$)).tw.

2. (proBNP adj5 (guide or monitor$ or target$ or predict$)).tw.

3. (NTproBNP adj5 (guide$ or monitor$ or target$ or predict$)).tw.

4. ((natriuretic peptide or natriuretic propeptide) adj5 (guide$ or monitor$ or target$ or predict$)).tw.

5. ((NTproBNP or Natriuretic Peptide or natriuretic propeptide or BNP or proBNP) adj5 (retest$ or serial or series)).tw.

6. ((NTproBNP or Natriuretic Peptide or natriuretic propeptide or BNP or proBNP) adj5 (manag$ or tailor$ or treat$ or therap$ or strateg$)).tw.

7. or/1-6

8. exp Heart Failure/

9. heart failure.tw.

10. cardiac failure.tw.

11. HT.tw.

12. CHF.tw.

13. or/8-12

14. Natriuretic Peptide, Brain/

15. Monitoring, Physiologic/

16. "Predictive Value of Tests"/

17. "Health Status Indicators"/

18. or/15-17

19. 14 and 18

20. (BNP adj5 (guide$ or monitor$ or target$ or predict$)).tw.

21. (proBNP adj5 (guide or monitor$ or target$ or predict$)).tw.

22. (NTproBNP adj5 (guide$ or monitor$ or target$ or predict$)).tw.

23. ((natriuretic peptide or natriuretic propeptide) adj5 (guide$ or monitor$ or target$ or predict$)).tw.

24. ((NTproBNP or Natriuretic Peptide or natriuretic propeptide or BNP or proBNP) adj5 (retest$ or serial or series)).tw.

25. ((NTproBNP or Natriuretic Peptide or natriuretic propeptide or BNP or proBNP) adj5 (manag$ or tailor$ or therap$ or strateg$)).tw.

26. or/20-25

27. 19 or 26

28. 13 and 27

29. randomized controlled trial.pt.

30. controlled clinical trial.pt.

31. randomized.ab.

32. placebo.ab.

33. drug therapy.fs.

34. randomly.ab.

35. trial.ab.

36. groups.ab.

37. or/29-36

38. exp animals/ not humans/

39. 37 not 38

40. 28 and 39

41. ("2014$" or "2015$" or "2016$").ed,yr.

42. 40 and 41

**OVID-EMBASE**

1. exp heart failure/

2. heart failure.tw.

3. cardiac failure.tw.

4. CHF.tw.

5. HF.tw.

6. or/1-5

7. brain natriuretic peptide/

8. monitoring/

9. predictive value/

10. "disease course"/

11. "symptom"/

12. "pathophysiology"/

13. patient monitoring/

14. biological monitoring/

15. hemodynamic monitoring/

16. or/8-15

17. 7 and 16

18. (BNP adj5 (guide$ or monitor$ or target$ or predict$)).tw.

19. (proBNP adj5 (guide or monitor$ or target$ or predict$)).tw.

20. (NTproBNP adj5 (guide$ or monitor$ or target$ or predict$)).tw.

21. ((natriuretic peptide or natriuretic propeptide) adj5 (guide$ or monitor$ or target$ or predict$)).tw.

22. ((NTproBNP or Natriuretic Peptide or natriuretic propeptide or BNP or proBNP) adj5 (retest$ or serial or series)).tw.

23. ((NTproBNP or Natriuretic Peptide or natriuretic propeptide or BNP or proBNP) adj5 (manag$ or tailor$ or treat$ or therap$ or strateg$)).tw.

24. or/18-23

25. 17 or 24

26. 6 and 25

27. random$.tw.

28. factorial$.tw.

29. (crossover$ or cross-over$).tw.

30. placebo$.tw.

31. (doubl$ adj blind$).tw.

32. (singl$ adj blind$).tw.

33. assign$.tw.

34. allocat$.tw.

35. volunteer$.tw.

36. Crossover Procedure/

37. Double-blind Procedure/

38. Randomized Controlled Trial/

39. Single-blind Procedure/

40. or/27-39

41. (animal/ or nonhuman/) not human/

42. 40 not 41

43. 26 and 42

44. limit 43 to embase

45. (2014$ or 2015$ or 2016$).em,yr.

46. 44 and 45 *[n=160]*

**Web of Science Core Collection**
Indexes=SCI-EXPANDED, SSCI, A&HCI, CPCI-S, CPCI-SSH, ESCI

# 1 TS=("heart failure" or "cardiac failure" or CHF or HF)

# 2 TS=("natriuretic peptide" NEAR target*) or TS=("natriuretic propeptide" NEAR target*)

# 3 TS=(BNP NEAR (guide* or monitor* or target* or predict*))

# 4 TS=(proBNP NEAR (guide or monitor* or target* or predict*))

# 5 TS=(NTproBNP NEAR (guide* or monitor* or target* or predict*))

# 6 TS=("natriuretic peptide" NEAR (guide* or monitor* or target* or predict*))

# 7 TS=("natriuretic propeptide" NEAR (guide* or monitor* or target* or predict*))

# 8 TS=((NTproBNP or "Natriuretic Peptide" or "natriuretic propeptide" or BNP or proBNP) NEAR retest*)

# 9 TS=((NTproBNP or "Natriuretic Peptide" or "natriuretic propeptide" or BNP or proBNP) NEAR serial*)

# 10 TS=((NTproBNP or "Natriuretic Peptide" or "natriuretic propeptide" or BNP or proBNP) NEAR series)

# 11 TS=((NTproBNP or "Natriuretic Peptide" or "natriuretic propeptide" or BNP or proBNP) NEAR (manag*))

# 12 TS=((NTproBNP or "Natriuretic Peptide" or "natriuretic propeptide" or BNP or proBNP) NEAR (tailor*))

# 13 TS=((NTproBNP or "Natriuretic Peptide" or "natriuretic propeptide" or BNP or proBNP) NEAR (therap*))

# 14 TS=((NTproBNP or "Natriuretic Peptide" or "natriuretic propeptide" or BNP or proBNP) NEAR (strateg*))

# 15 #14 OR #13 OR #12 OR #11 OR #10 OR #9 OR #8 OR #7 OR #6 OR #5 OR #4 OR #3 OR #2

# 16 #15 AND #1

# 17 TS=(random* or trial or placebo* or groups (double same blind*) or (single same blind*))

# 18 #17 AND #16

#19 TS=(((clinical near trial* or crossover or cross over) or ((single* or doubl* or trebl* or tripl*) near (blind* or mask* or dummy)) or (singleblind* or doubleblind* or trebleblind* or tripleblind* or placebo* or random*))) or TI=(((clinical near trial* or crossover or cross over) or ((single* or doubl* or trebl* or tripl*) near (blind* or mask* or dummy)) or (singleblind* or doubleblind* or trebleblind* or tripleblind* or placebo* or random*)))

**The Cochrane Library**

#1 MeSH descriptor: [Heart Failure] explode all trees

#2 "heart failure"

#3 "cardiac failure"

#4 CHF or HF:ab (Word variations have been searched)

#5 #1 or #2 or #3 or #4

#6 MeSH descriptor: [Natriuretic Peptide, Brain] this term only

#7 (BNP near/5 (guide* or monitor* or target* or predict*))

#8 (NTproBNP near/5 (guide* or monitor* or target* or predict*))

#9 (("natriuretic peptide") near/5 (guide* or monitor* or target* or predict*))

#10 ((NTproBNP or "Natriuretic Peptide" or "natriuretic propeptide" or BNP or proBNP) near/5 (retest* or serial or series))

#11 ("natriuretic propeptide" near/5 (guide* or monitor* or target* or predict*))

#12 (NTproBNP or "Natriuretic Peptide" or "natriuretic propeptide" or BNP or proBNP):ti

#13 (NTproBNP or "Natriuretic Peptide" or "natriuretic propeptide" or BNP or proBNP) near/5 (manag* or tailor* or therap* or strateg*)

#14 (proBNP near/5 (guide* or monitor* or target* or predict*))

#15 (#6 or #7 or #8 or #9 or #10 or #11 or #12 or #13 or #14)

#16 #5 and #15

#17 (2014 or 2015 or 2016)

#18 #16 and #17
