# Supplementary material for: B-type natriuretic peptide-guided therapy for heart failure (HF): a systematic review and meta-analysis of individual participant data (IPD) and aggregate data
Source: Syst Rev. 2018 Jul 31;7:112. doi: 10.1186/s13643-018-0776-8 (PMC6069819; doi:10.1186/s13643-018-0776-8)
Supplement: Supplementary file 2 — Appendix 2. Risk of bias. (DOCX 132 kb) [file 13643_2018_776_MOESM2_ESM.docx]

Appendix 2

Risk of bias

Review authors’ judgements about each risk of bias item presented as percentages for all included studies.

9/14 RCTs (RCTs that contributed aggregate data) were used to assess risk of bias from incomplete outcome data and selective outcome reporting. For all other risk of bias domains all 14 RCTs were used.

Review authors’ judgements about each risk of bias item presented for all the included studies

| Study | Selection bias | | Performance bias | | | | Detection bias | | | | Attrition bias | | | | Reporting bias | Other bias |
| --- | --- | --- | --- | --- | --- | --- | --- | --- | --- | --- | --- | --- | --- | --- | --- | --- |
|  |  |  | Blinding of participants and personnel | | | | Blinding of outcome assessment | | | | Incomplete outcome data^§^ | | | | Selective reporting^§^ | Other sources of bias |
|  | Random sequence generation | Allocation concealment | All-cause mortality | Cause specific mortality^*^ | Harms^**^ | Quality of life | All-cause mortality | Cause specific mortality^*^ | Harms^**^ | Quality of life | All-cause mortality | Cause specific mortality^*^ | Harms^**^ | Quality of life |  |  |
| Anguita [20] | Unclear | Unclear | High | High | High | Unclear | Low | High | High | Unclear | - | - | - | - | - | Low |
| Northstar [27] | Low | Unclear | High | High | High | High | Low | Low | Low | High | - | - | - | - | - | Unclear |
| Shochat [32]  *Published as abstract only* | Unclear | Unclear | Unclear | Unclear | Unclear | Unclear | Low | Unclear | Unclear | Unclear | - | - | - | - | - | Unclear |
| Starbrite [31] | Low | Low | High | Unclear | High | Unclear | Low | Unclear | High | Unclear | - | - | - | - | - | Low |
| Upstep [24] | Low | Unclear | High | High | High | Unclear | Low | Low | Low | Unclear | - | - | - | - | - | Unclear |
| Christchurch Pilot [29] | Unclear | Unclear | Low | Unclear | Low | Low | Low | Unclear | Low | Low | Low | Unclear | Low | Unclear | Unclear | Low |
| Time-CHF^*^ [26, 33] | Low | Low | High | High | High | Low | Low | Low | Low | Low | Low | Low | Low | Unclear | Low | Low |
| Berger [21] | Low | Low | High | Unclear | High | Unclear | Low | Unclear | Low | Unclear | Unclear | Unclear | Unclear | Unclear | Unclear | Low |
| Prima [22] | Unclear | Unclear | High | High | High | Unclear | Low | Low | Low | High | Low | Low | Low | Unclear | Low | Low |
| Signal-HF [25] | Unclear | Unclear | High | High | High | Unclear | Low | High | High | Unclear | Low | Low | Low | Unclear | High | Unclear |
| Battlescarred [28] | Low | Unclear | Low | Unclear | Low | Low | Low | Unclear | Low | Low | Unclear | Unclear | Unclear | Unclear | Low | Low |
| Stars-BNP [23] | Unclear | Unclear | High | High | High | Unclear | Low | Unclear | Low | Unclear | Low | Low | Low | Unclear | Unclear | Unclear |
| Protect [30] | Low | Unclear | High | High | High | High | Low | Low | Low | High | Low | Low | Low | Low | Low | Unclear |
| Guide-IT [11] | Low | Unclear | High | High | High | Unclear | Low | Low | Low | Unclear | Low | Low | Low | Unclear | Low | Low |

^§^ Only trials that provided aggregate data were assessed for risk of bias from incomplete outcome data and selective outcome reporting

^*^Death related to HF and death from any cardiovascular cause ^**^All-cause hospital admission, hospital admission for HF, other serious adverse events
