# Supplementary material for: B-type natriuretic peptide-guided therapy for heart failure (HF): a systematic review and meta-analysis of individual participant data (IPD) and aggregate data
Source: Syst Rev. 2018 Jul 31;7:112. doi: 10.1186/s13643-018-0776-8 (PMC6069819; doi:10.1186/s13643-018-0776-8)

Appendix 3

Funnel plots

Funnel plot for the primary outcome of all-cause mortality


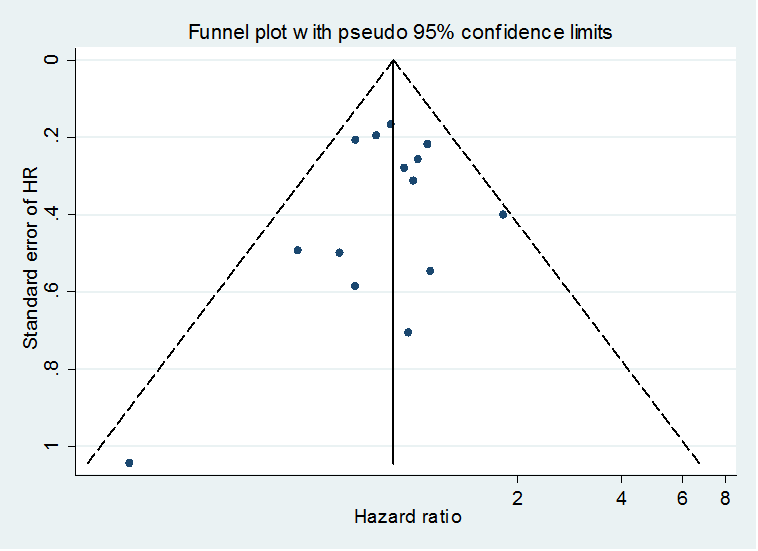


Funnel plot for the secondary outcome of HF hospitalization


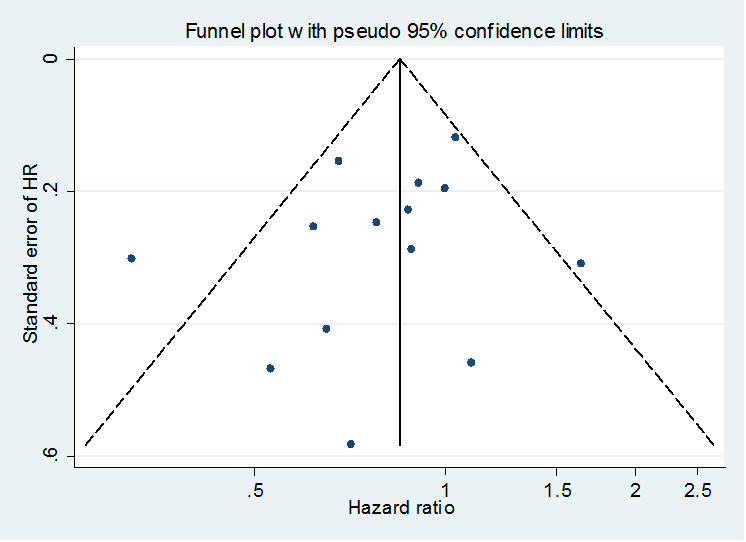

Supplement: Supplementary file 3 — Appendix 3. Funnel plots. (DOCX 41 kb) [file 13643_2018_776_MOESM3_ESM.docx]
