# Supplementary material for: B-type natriuretic peptide-guided therapy for heart failure (HF): a systematic review and meta-analysis of individual participant data (IPD) and aggregate data
Source: Syst Rev. 2018 Jul 31;7:112. doi: 10.1186/s13643-018-0776-8 (PMC6069819; doi:10.1186/s13643-018-0776-8)
Supplement: Supplementary file 4 — Appendix 4. Subgroup analyses. (DOCX 851 kb) [file 13643_2018_776_MOESM4_ESM.docx]

Appendix 4

Subgroup analyses

Subgroup analysis younger vs older age (<75 years vs ≥75 years). All-cause mortality: unadjusted individual hazards ratios (HR) with 95% confidence intervals (CI) for four studies providing individual patient data (IPD) and seven studies included in a previous meta-analysis (aggregate).

Age <75 years


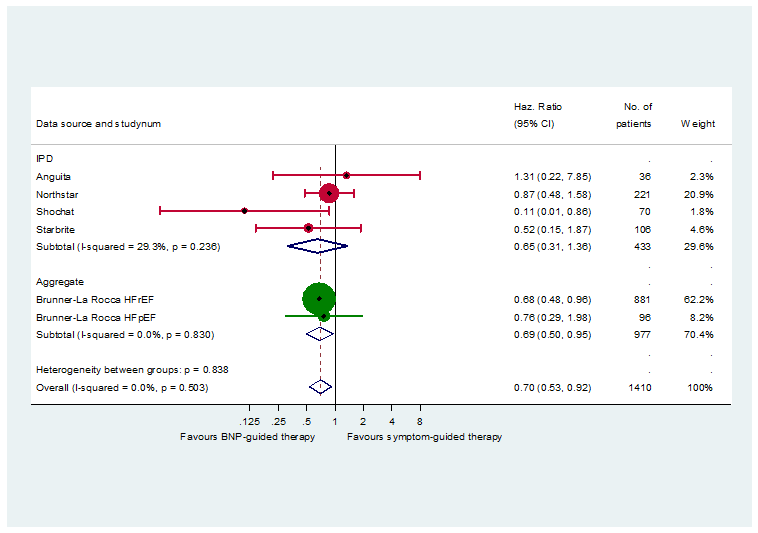


Age ≥75 years


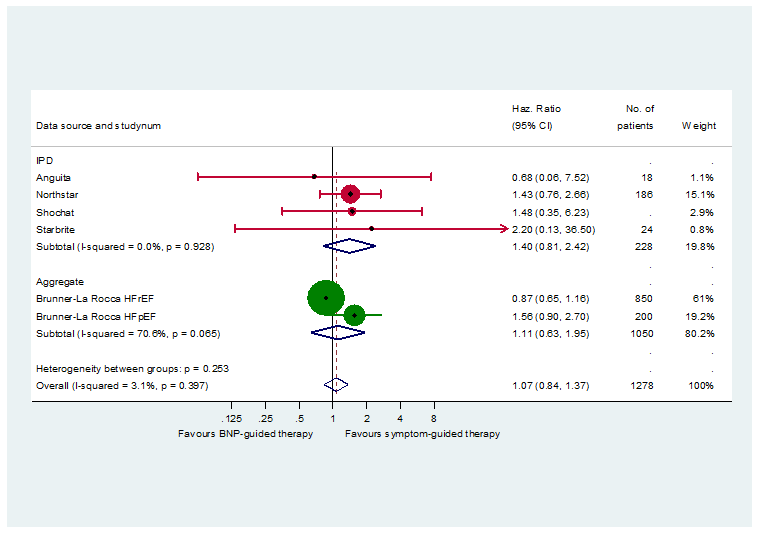


Subgroup analysis younger vs older age (<75 years vs ≥75 years). All-cause hospitalisation: unadjusted individual hazards ratios (HR) with 95% confidence intervals (CI) for five studies providing individual patient data (IPD) and one study providing aggregate data. Meta-analysis HR (95% CI) presented both within IPD and aggregate data sources, and overall.

Age <75 years


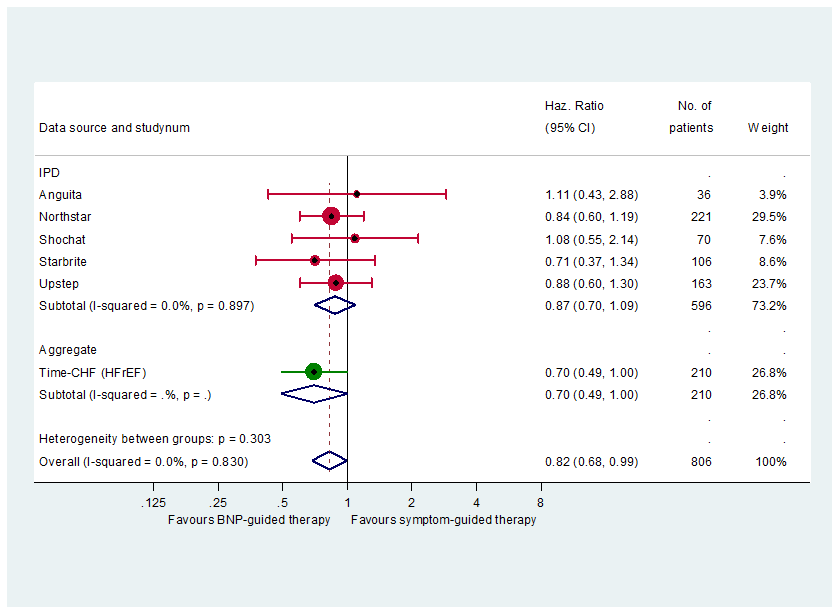


Age ≥75 years


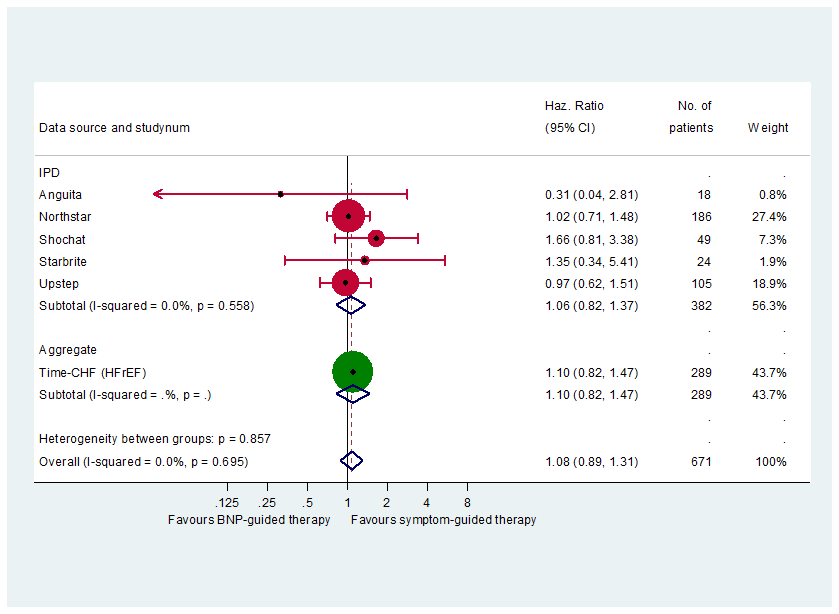


Subgroup analysis age (<75 years vs ≥75 years). Heart failure hospitalisation: unadjusted individual hazards ratios (HR) with 95% confidence intervals (CI) for fourstudies providing individual patient data (IPD) and one study providing aggregate data. Meta-analysis HR (95% CI) presented both within IPD and aggregate data sources, and overall.

Age <75 years


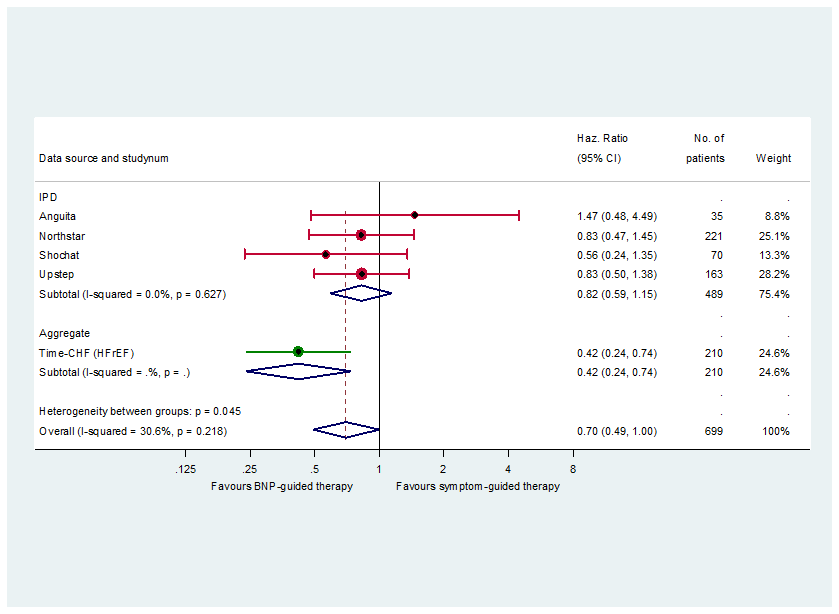


Age ≥75 years


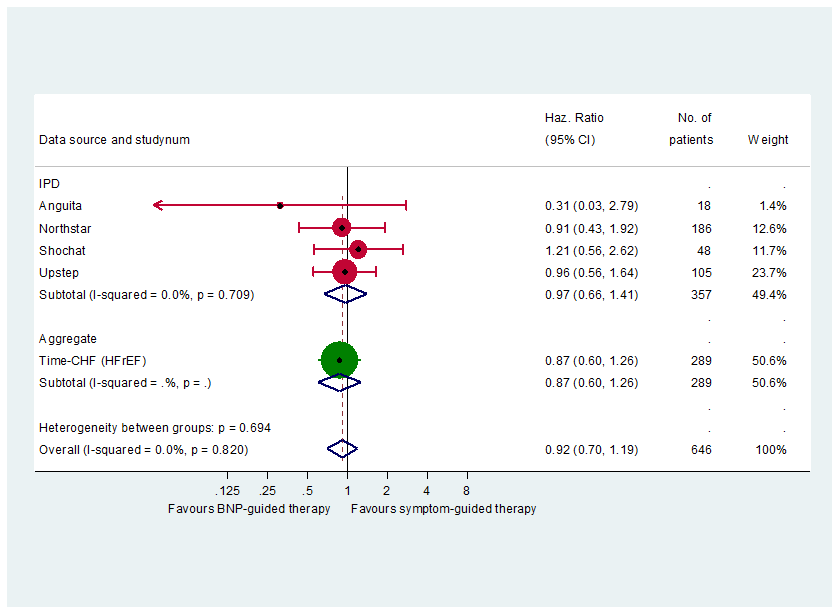


Subgroup analysis men vs women. All-cause mortality: unadjusted individual hazards ratios (HR) with 95% confidence intervals (CI) for five studies providing individual patient data (IPD) and one study providing aggregate data. Meta-analysis HR (95% CI) presented both within IPD and aggregate data sources, and overall.

Men


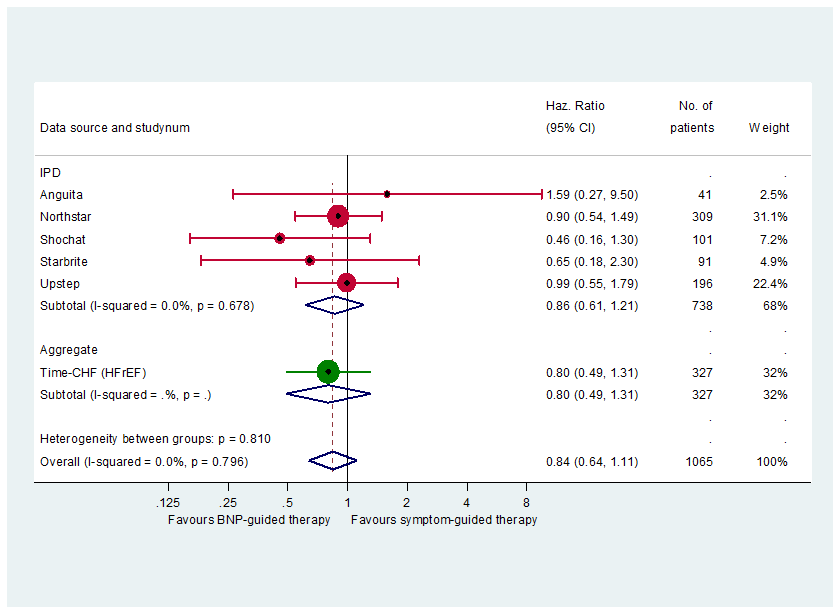


Women


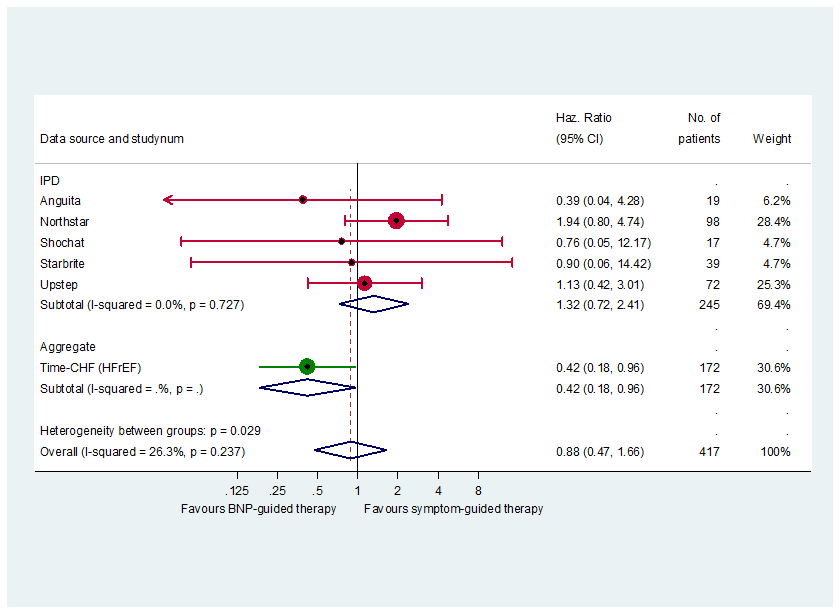


Subgroup analysis men vs women. All-cause hospitalisation: unadjusted individual hazards ratios (HR) with 95% confidence intervals (CI) for five studies providing individual patient data (IPD) and one study providing aggregate data. Meta-analysis HR (95% CI) presented both within IPD and aggregate data sources, and overall.

Men


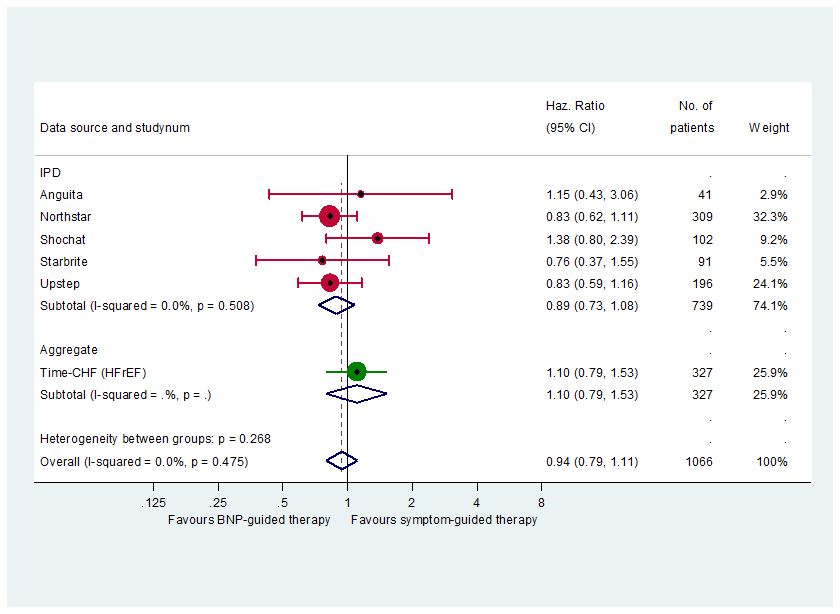


Women

*
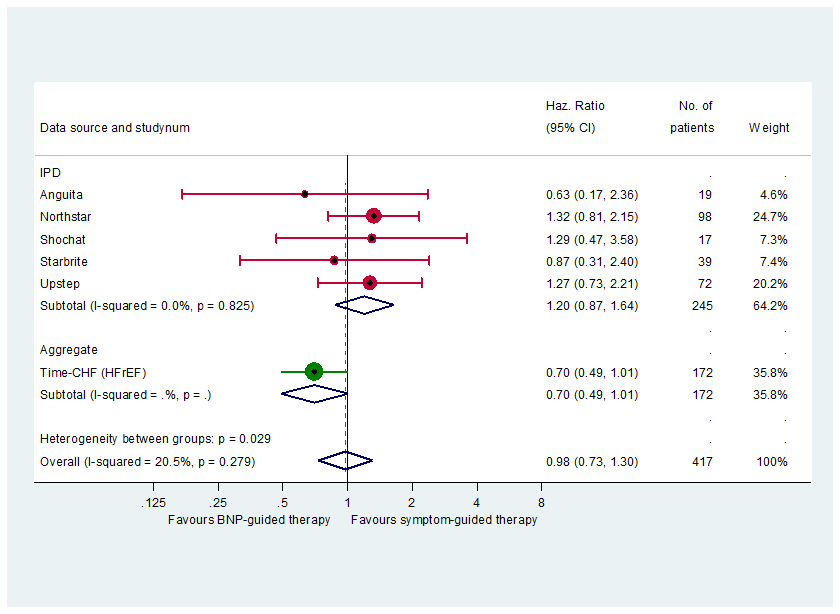
*

Subgroup analysis men vs women. Heart failure hospitalisation: unadjusted individual hazards ratios (HR) with 95% confidence intervals (CI) for four studies providing individual patient data (IPD) and one study providing aggregate data. Meta-analysis HR (95% CI) presented both within IPD and aggregate data sources, and overall.

Men

*
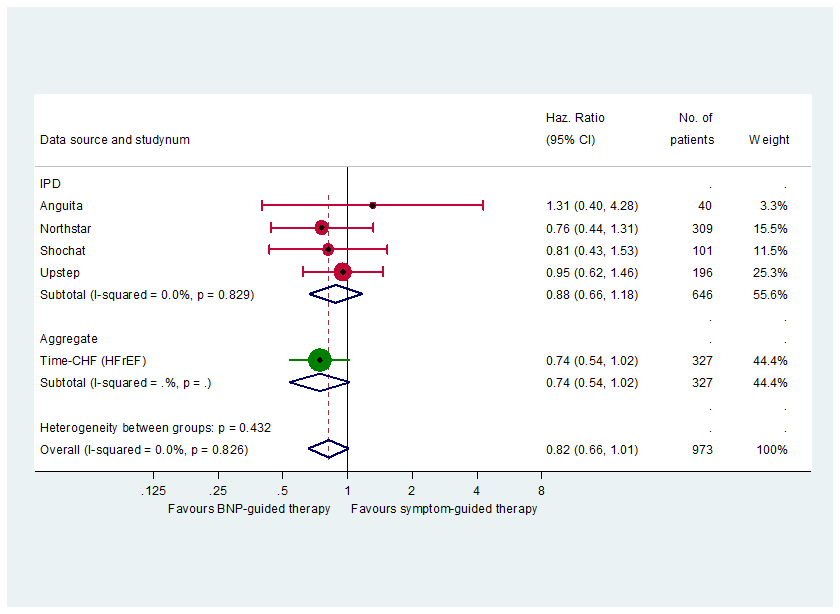
*

Women

*
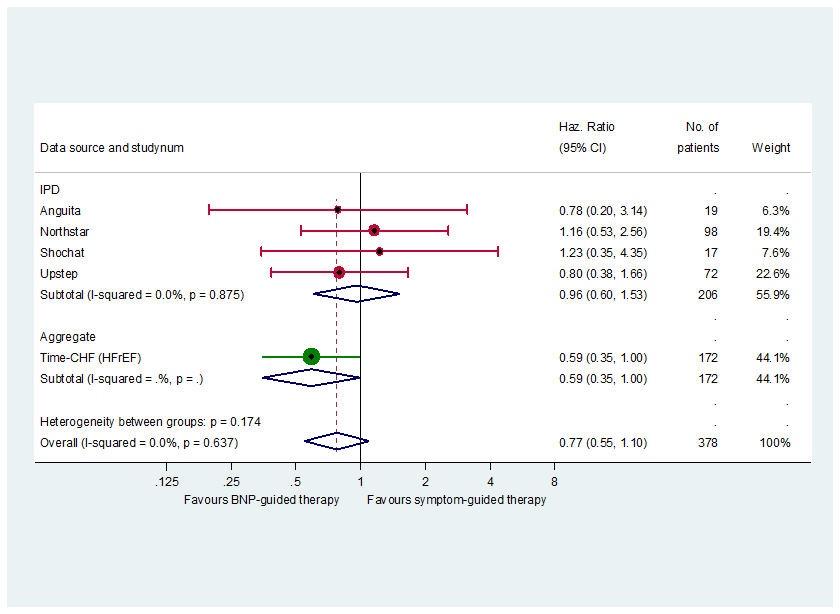
*

Subgroup analysis New York Heart Association class (class I/II vs class III/IV). All-cause mortality: unadjusted individual hazards ratios (HR) with 95% confidence intervals (CI) for three studies providing individual patient data (IPD), with meta-analysis HR and 95% CI.

NYHA class I/II
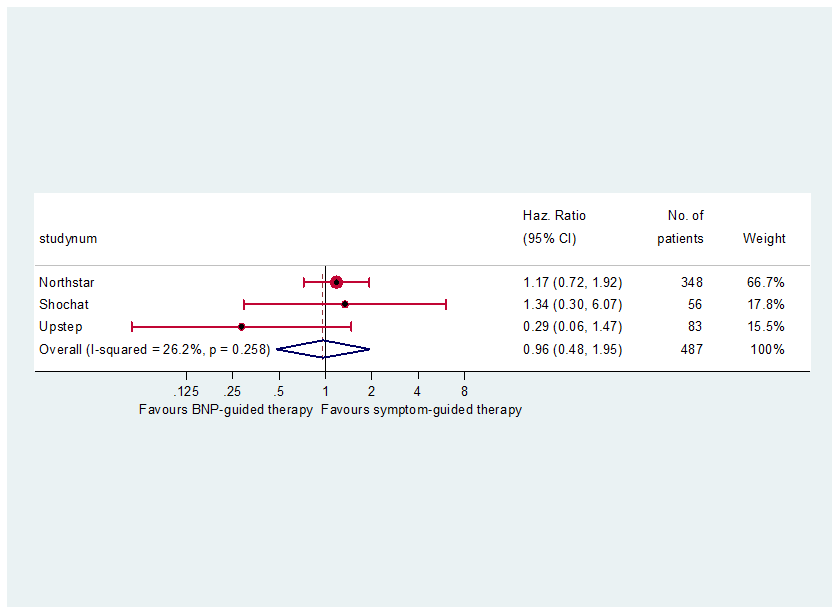


NYHA class III/IV

*
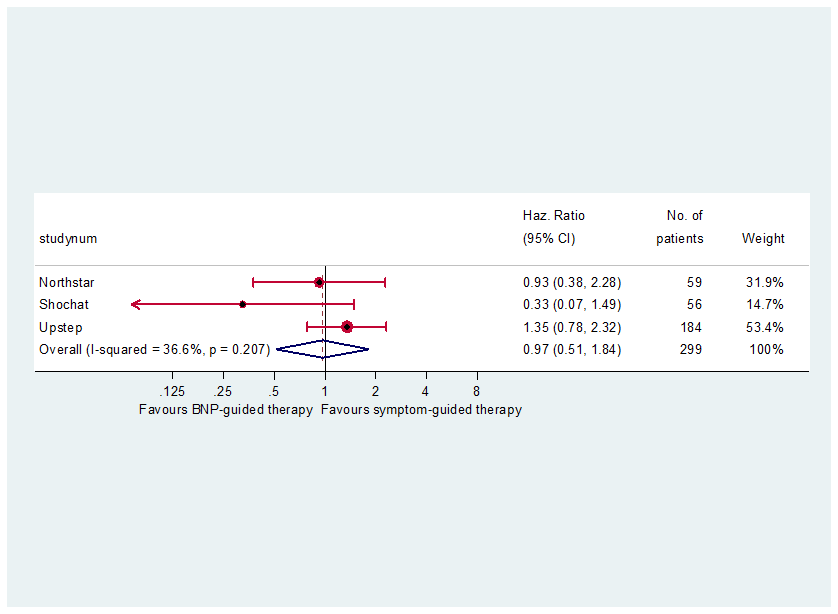
*

Subgroup analysis New York Heart Association class (class I/II vs class III/IV). All-cause hospitalisation: unadjusted individual hazards ratios (HR) with 95% confidence intervals (CI) for four studies providing individual patient data (IPD), with meta-analysis HR and 95% CI.

NYHA class I/II
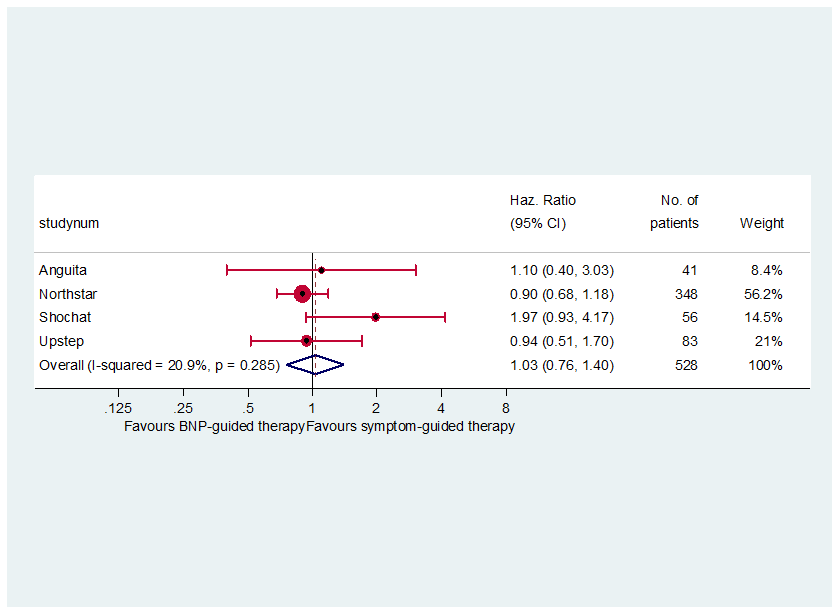


NYHA class III/IV

*
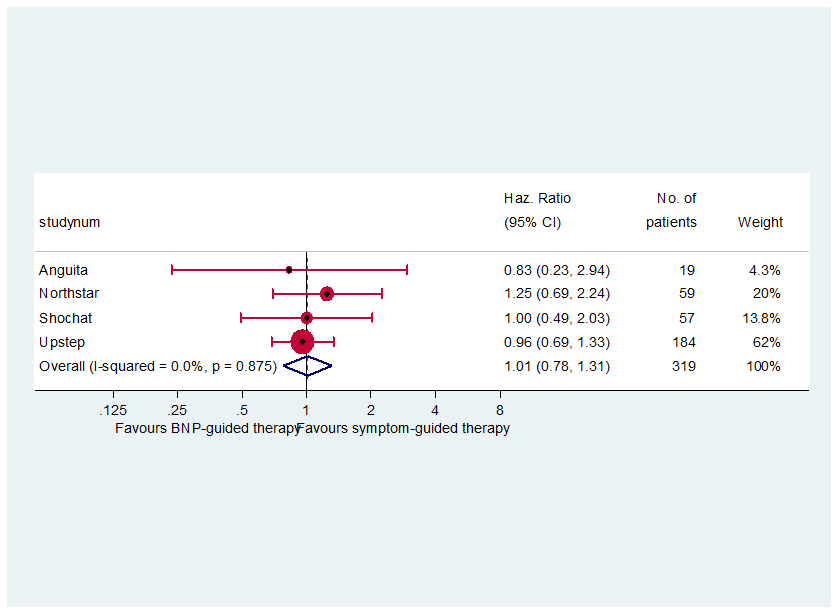
*

Subgroup analysis New York Heart Association class (class I/II vs class III/IV). Heart failure hospitalisation: unadjusted individual hazards ratios (HR) with 95% confidence intervals (CI) for four studies providing individual patient data (IPD), with meta-analysis HR and 95% CI.

NYHA class I/II

*
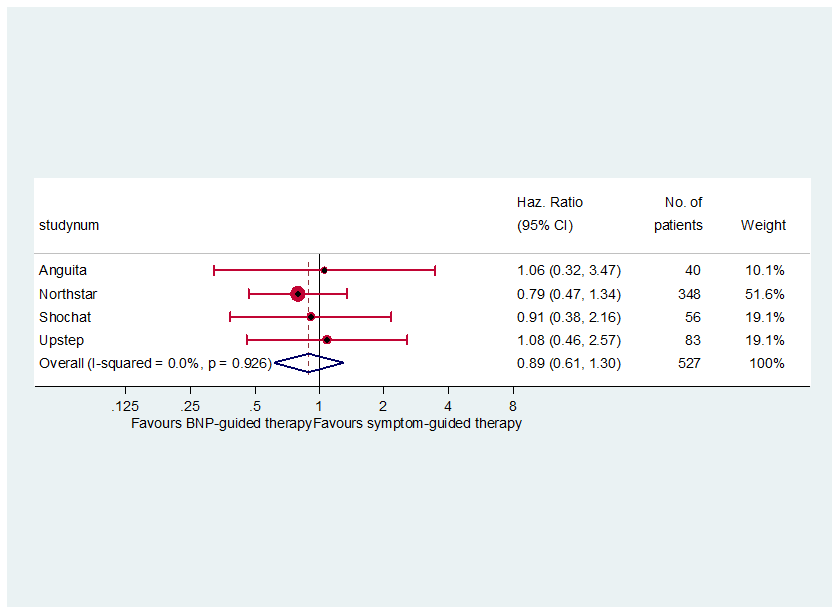
*

NYHA class III/IV

*
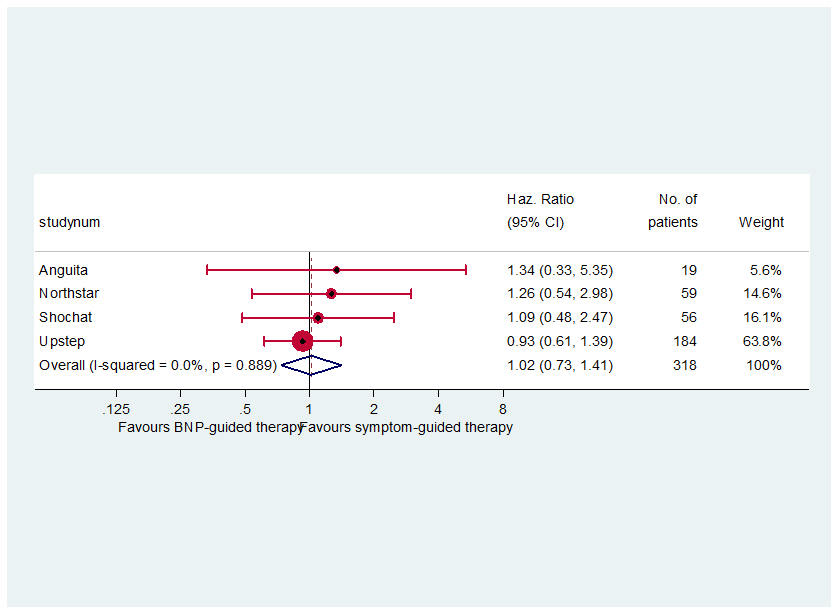
*

Subgroup analysis left ventricular ejection fraction (≤40% vs >40% for IPD studies and ≤45% vs >45% for aggregate studies, except for Guide-It which included patients with LVEF ≤40%). All-cause mortality: unadjusted individual hazards ratios (HR) with 95% confidence intervals (CI) for four studies providing individual patient data (IPD) and six studies included in a previous IPD meta-analysis (aggregate^*^).

Reduced LVEF (HFrEF)


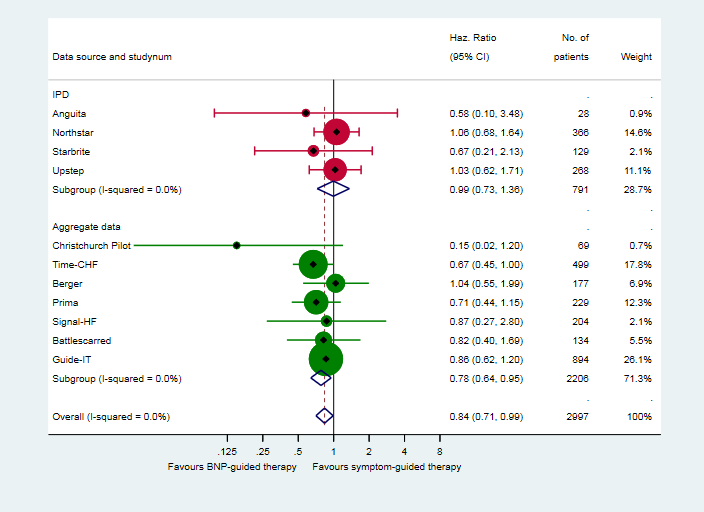


Preserved LVEF (HFpEF)


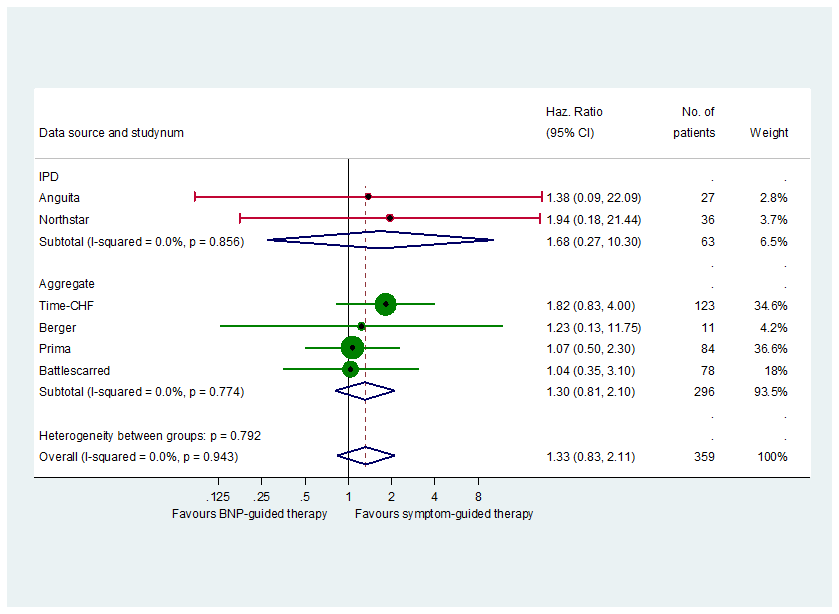


^*^ The individual estimates shown in the aggregate subgraph were reported previously [17].

Subgroup analysis left ventricular ejection fraction (≤40% vs >40% for IPD studies and ≤45% vs >45% for aggregate studies). All-cause hospitalisation: unadjusted individual hazards ratios (HR) with 95% confidence intervals (CI) for four studies providing individual patient data (IPD) and one study providing aggregate data, with meta-analysis HR and 95% CI.

*Reduced LVEF (HFrEF)*


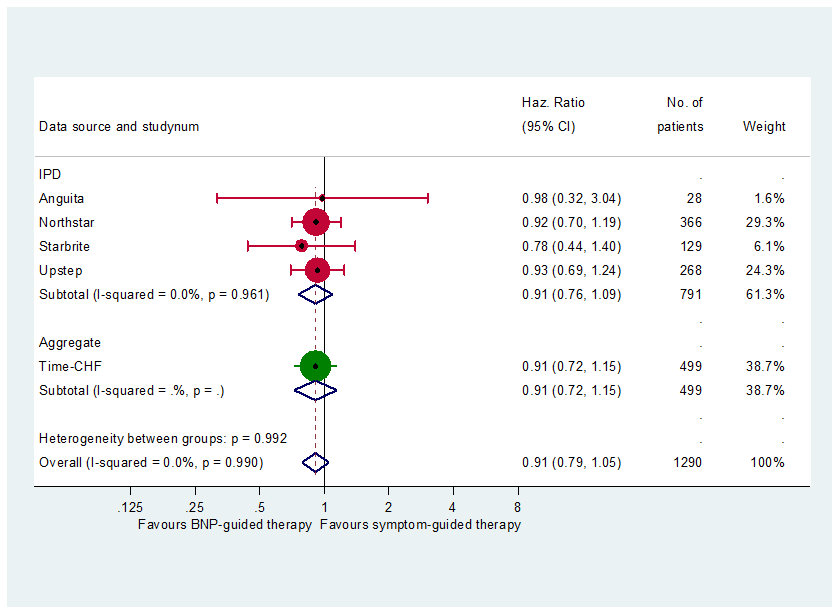


Preserved LVEF (HFpEF)

*
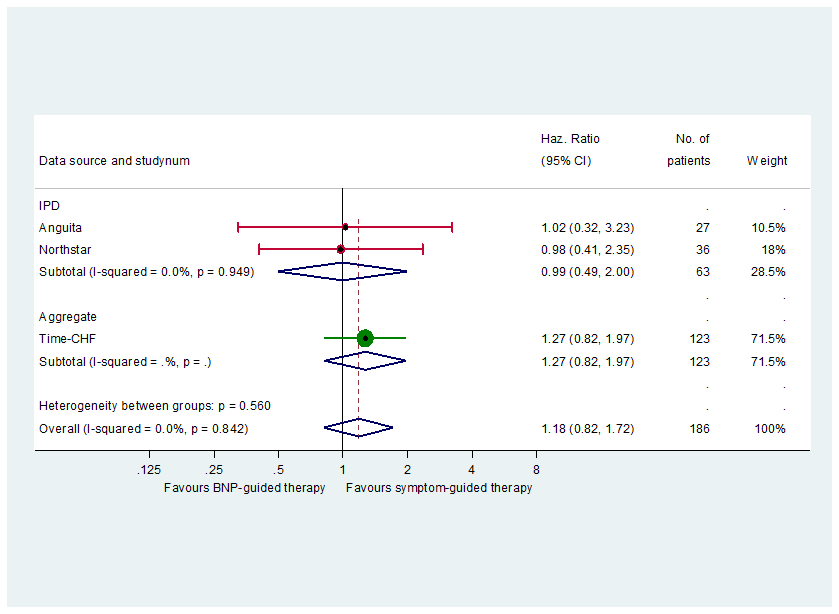
*

Subgroup analysis left ventricular ejection fraction (≤40% vs >40% for IPD studies and ≤45% vs >45% for aggregate studies, except for Guide-It which included patients with LVEF ≤40%). Heart failure hospitalisation: unadjusted individual hazards ratios (HR) with 95% confidence intervals (CI) for two studies providing individual patient data (IPD), and onestudies included in a previous IPD meta-analysis (aggregate).

*Reduced LVEF (HFrEF)*


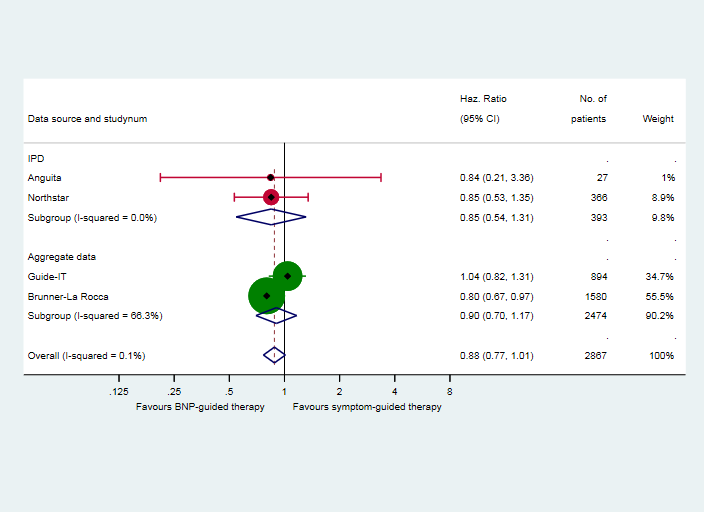


Preserved LVEF (HFpEF)


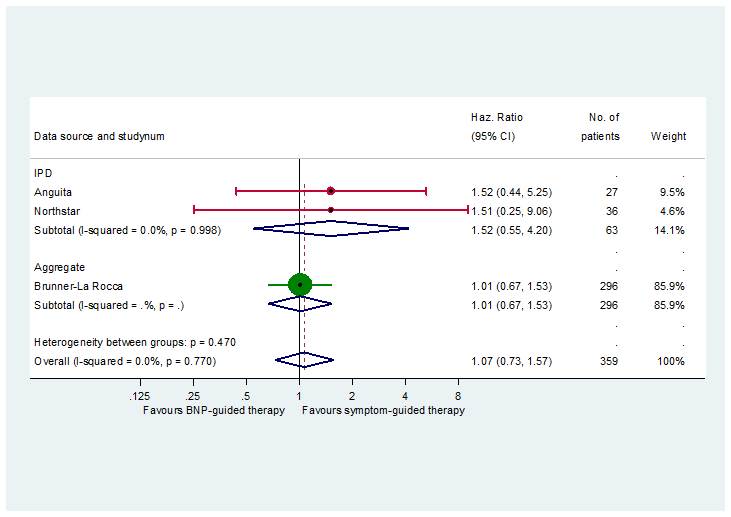


Subgroup analysis diabetic vs non-diabetic. All-cause mortality: unadjusted individual hazards ratios (HR) with 95% confidence intervals (CI) for four studies providing individual patient data (IPD) and one study providing aggregate data. Meta-analysis HR (95% CI) presented both within IPD and aggregate data sources, and overall.

Non-diabetic


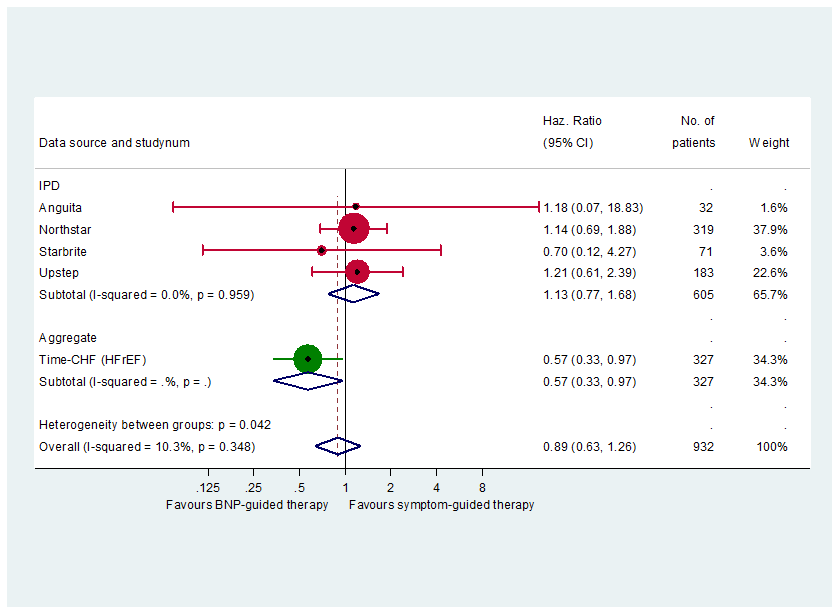


*Diabetic*


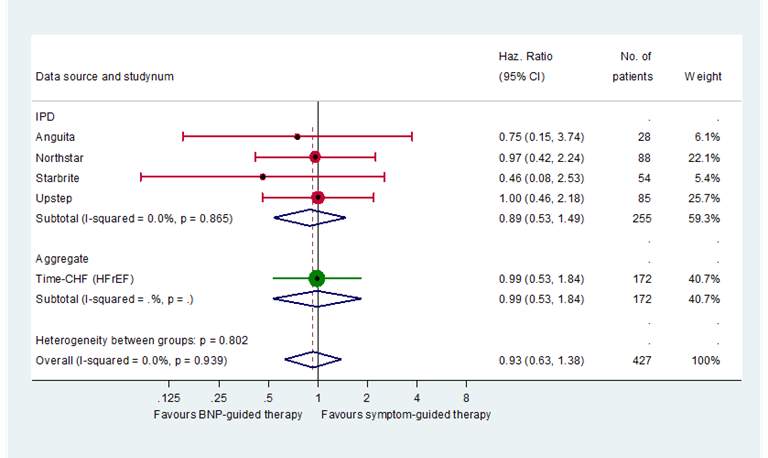


Subgroup analysis diabetic vs non-diabetic. All-cause hospitalisation: unadjusted individual hazards ratios (HR) with 95% confidence intervals (CI) for four studies providing individual patient data (IPD) and one study providing aggregate data. Meta-analysis HR (95% CI) presented both within IPD and aggregate data sources, and overall.

Non-diabetic
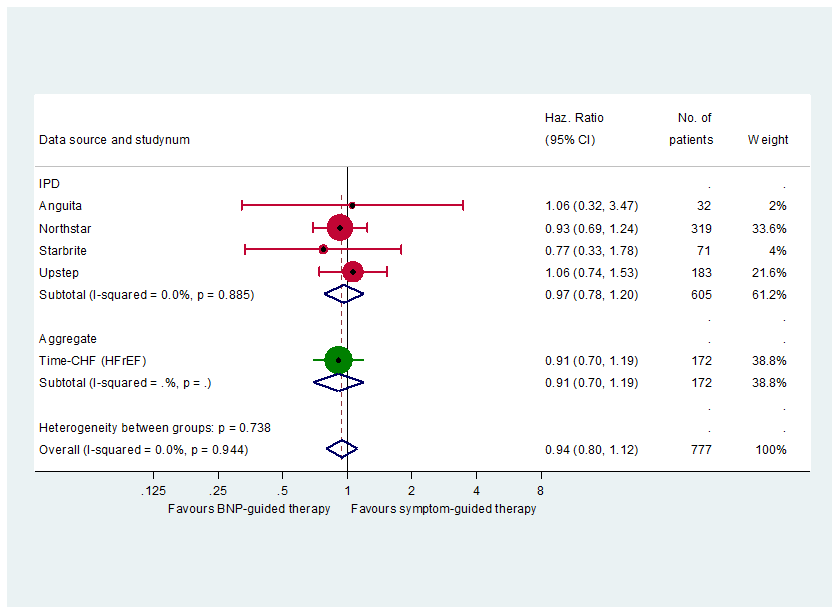


Diabetic

*
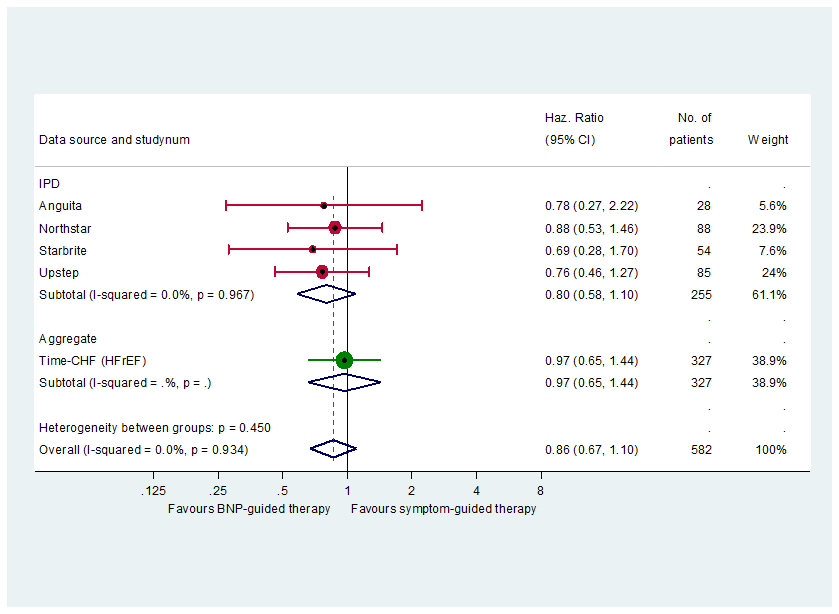
*

Subgroup analysis diabetic vs non-diabetic. Heart failure hospitalisation: unadjusted individual hazards ratios (HR) with 95% confidence intervals (CI) for three studies providing individual patient data (IPD) and one study providing aggregate data. Meta-analysis HR (95% CI) presented both within IPD and aggregate data sources, and overall.

Non-diabetic
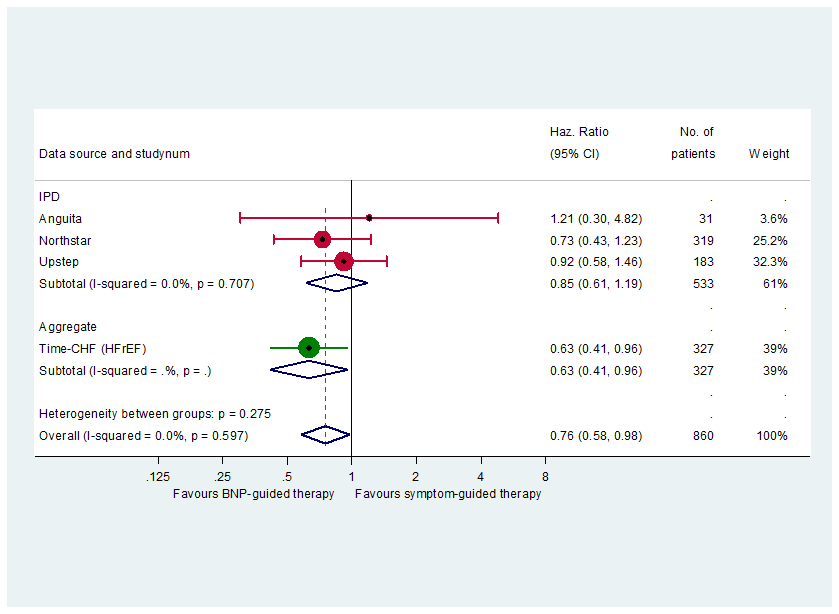


Diabetic


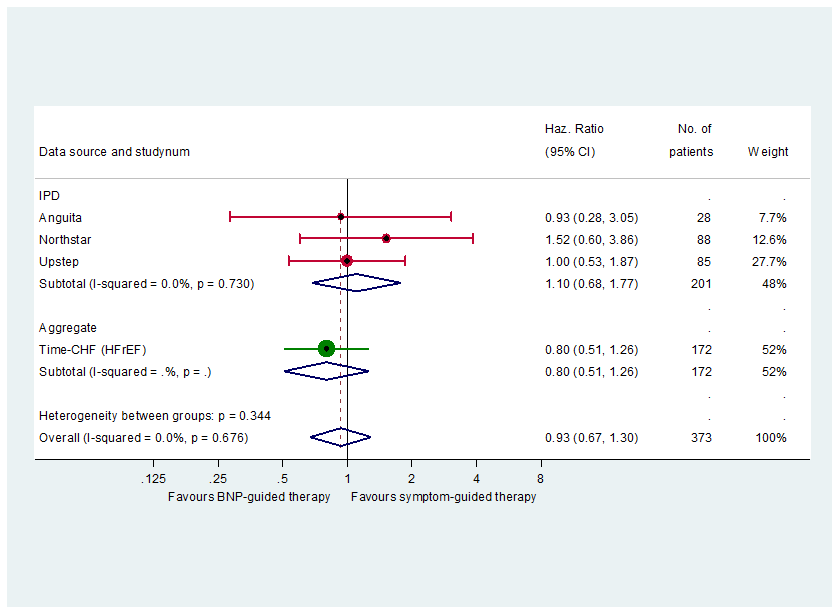


Subgroup analysis high vs low BNP at baseline. All-cause mortality: unadjusted individual hazards ratios (HR) with 95% confidence intervals (CI) for four studies providing individual patient data (IPD) and one study providing aggregate data. Meta-analysis HR (95% CI) presented both within IPD and aggregate data sources, and overall.

BNP ≤ median


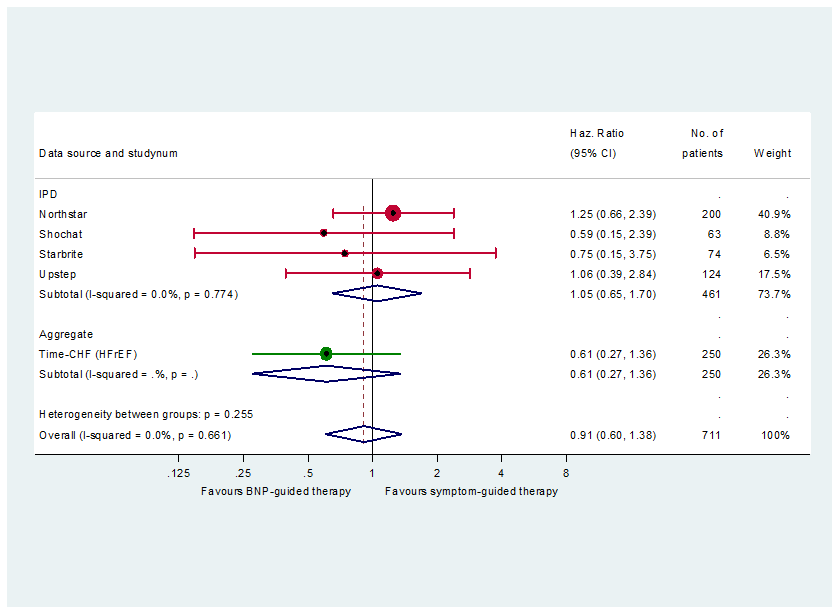


BNP > median
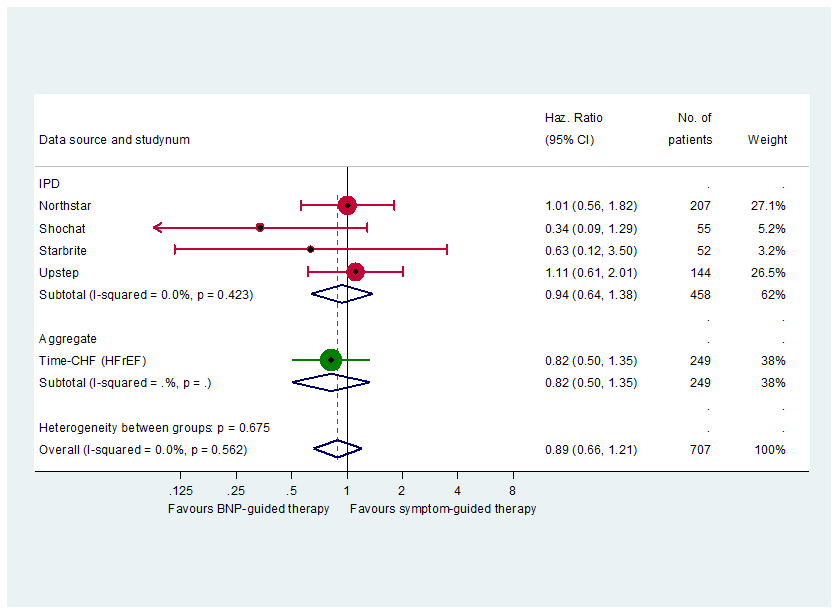


Subgroup analysis high vs low BNP at baseline. All-cause hospitalisation: unadjusted individual hazards ratios (HR) with 95% confidence intervals (CI) for four studies providing individual patient data (IPD) and one study providing aggregate data. Meta-analysis HR (95% CI) presented both within IPD and aggregate data sources, and overall.

BNP ≤ median
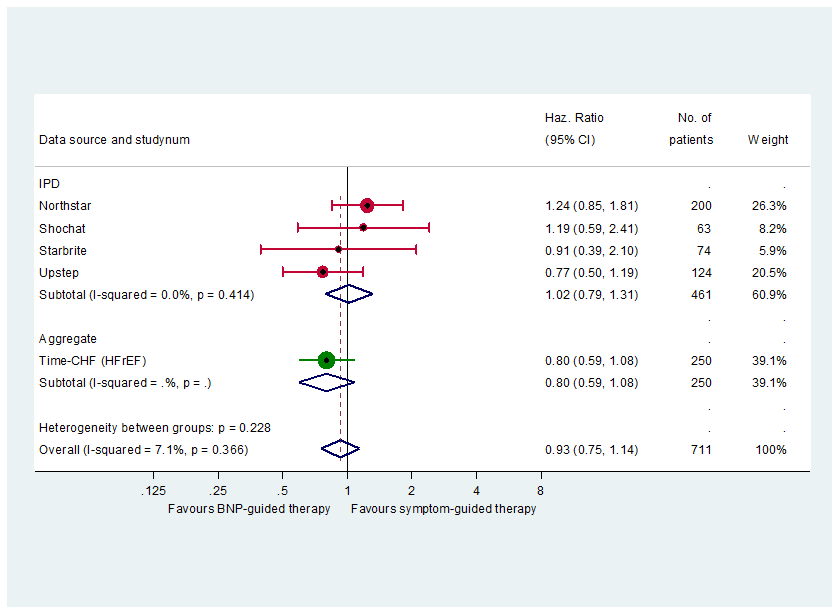


BNP > median

*
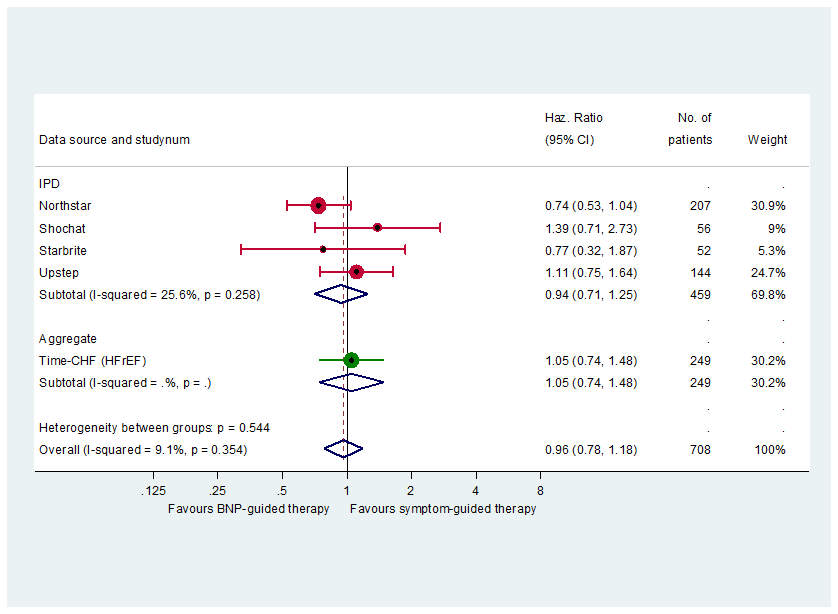
*

Subgroup analysis high vs low BNP at baseline. Heart failure hospitalisation: unadjusted individual hazards ratios (HR) with 95% confidence intervals (CI) for three studies providing individual patient data (IPD) and one study providing aggregate data. Meta-analysis HR (95% CI) presented both within IPD and aggregate data sources, and overall.

BNP ≤ median


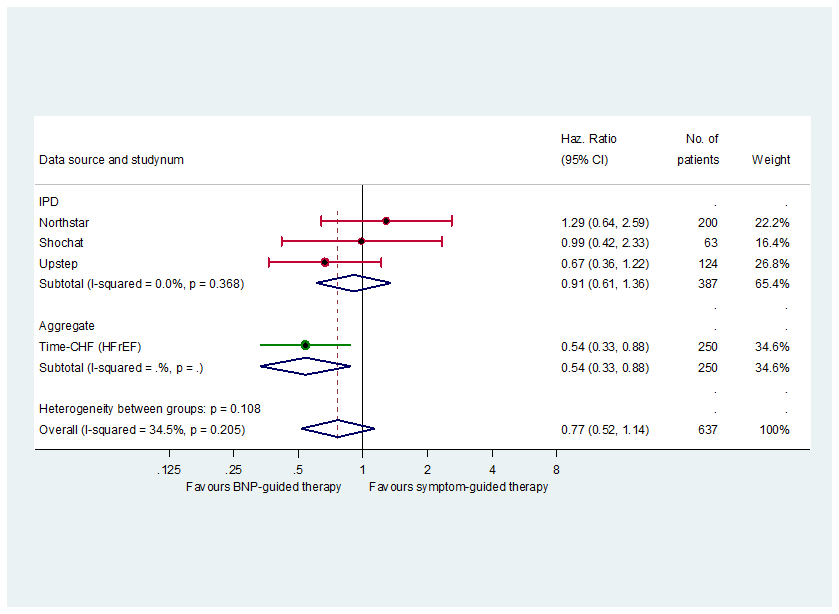


BNP > median

*
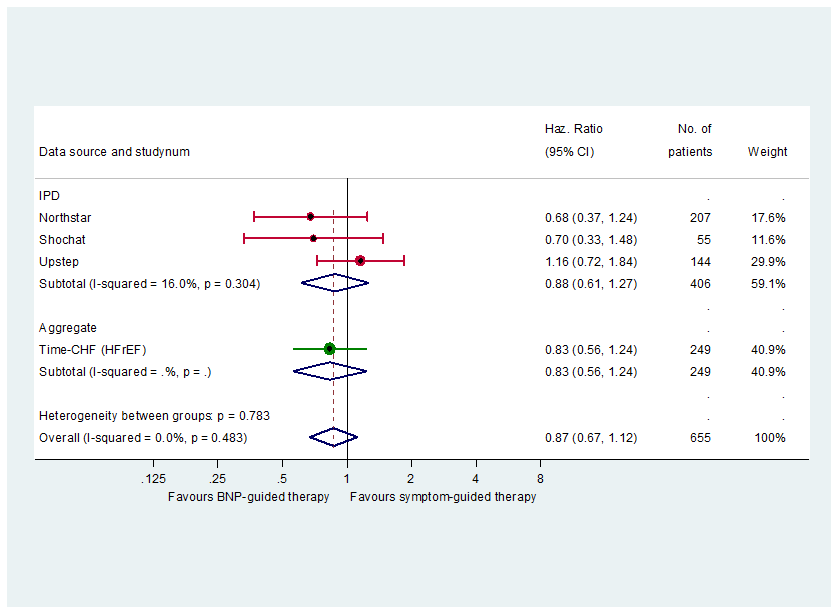
*
